# A methodology for integrating AI into embodied human intelligence for the performance of complex tasks

**DOI:** 10.3389/frai.2026.1715898

**Published:** 2026-03-04

**Authors:** Tamim Ahmed, Thanassis Rikakis

**Affiliations:** 1 Department of Biomedical Engineering, University of Southern California, Los Angeles, CA, United States; 2 Human Technology Interaction & Health Innovation, USC Iovine and Young Academy, University of Southern California (USC), Los Angeles, CA, United States

**Keywords:** augmented intelligence, complex tasks, computational ensemble, Dynamic Bayesian Network, embodied cognition, gated pipeline, human-AI collaboration

## Abstract

We propose a theory and methodology for designing human-artificial intelligence (AI) collaboration in complex, embodied tasks. The theory distinguishes human embodied intelligence from computational intelligence and identifies synergies in which AI enhances—rather than replicates or replaces—human performance. We represent observable structures of expert performance as a nested network with four interdependent layers: *Environment* (space and tools), *Activity* (what is done), *Goals* (what is aimed for), and *Meaning* (how performance is interpreted), all connected by dynamic four-layer edges. A bidirectional Dynamic Bayesian Network (DBN) computes this representation across temporal scales: instants, actions, complete performances, and sequences. The DBN informs the design of digital tools (from sensors to data structures and AI modules) that capture human performance and extract features, descriptors, and predictions that enhance the observability and analysis of performance. During task performance, a *top-down pass* predicts expert orientation—current goals and interpretations—and drives a search policy that selects where to look. A *bottom-up pass* processes action-conditioned computational observations and filters them through a gated pipeline to produce new candidates for four-layer connectivity (c4). After expert validation, candidates update the network, sharpening DBN posteriors, reducing entropy, and thereby enhancing human performance. We instantiated this framework in automated physical rehabilitation assessment through a 12-month deployment with 10 clinicians and 105 stroke survivors. Co-design cycles developed and enriched a four-layer DBN representation of rehabilitation assessment and informed the design of a computational ensemble for automated assessment. The computational ensemble achieved 90.8% agreement with clinicians at the exercise level, 93.1% at the segment level, and 90.6% at the movement quality level. Clinicians validated automated assessments at high rates and reported improved confidence and efficiency when leveraging ensemble insights for therapy assessment and planning. This portable methodology and theory can be applied to the embodied performance of complex tasks across multiple applications.

## Introduction

This study proposes a methodology for exploring and structuring collaborations between human embodied intelligence and artificial intelligence. The methodology focuses on applying artificial intelligence (AI) to complex embodied tasks with formalized, observable structures. The complexity of these tasks requires highly developed embodied intelligence and offers significant opportunities for human-AI collaboration, where technology complements rather than replaces people. The observable and formalized elements of the tasks provide an entry point for computational modeling that can facilitate meaningful human-AI collaboration. The targeted tasks can range from designing a smart product to performing surgery in a modern operating room or conducting a search-and-rescue operation using autonomous agents.

In the background section, we present prior work on embodied human intelligence that links cognitive development to sensorimotor activity and to task performance in the wild. Within this context, human symbol creation and processing are strongly related to the extraction of meaning from interactions in the wild to inform future interactions. Therefore, human symbol processing is well differentiated from, and potentially synergistic with, computational symbol processing. Computation focuses on extracting relationships among symbols rather than on relations between symbols and embodied needs and actions. Humans improve their activities in the wild through constant learning and the development of tools. Our methodology approaches artificial intelligence as a means (a tool) for extending explicit symbol processing within complex embodied actions by humans to inform and enrich these actions.

In the methodology section, we represent human embodied performance in complex tasks as a nested network. The nested network comprises the following layers (listed from containing to contained): environment, human embodied activity, embodied goals, and meaning. The components and interconnections of the nested network are complex and not fully observable because many are embodied and tacit. Furthermore, they change as interaction drives human learning and as the environment evolves. We then facilitate human-AI collaboration by computationally modeling the nested network as a bidirectional Dynamic Bayesian Network (DBN). The DBN (i) *translates* the expert's symbols and routines (recorded in the nested network) into top–down information needs and bottom–up probabilistic updates; (ii) *informs* the design of digital tools (from sensors to data structures and AI engines) that can track and analyze task performance; (iii) *produces* symbolic descriptors and related calibrated likelihoods that increase the coherence and observability of the network and enhance the probability of improved performance; and (iv) *assesses* enhanced human performance and computational design in an integrative manner. As with all human-made tools, the inclusion of computational tools and intelligence in an activity will alter the embodied activity and its environment, thereby informing subsequent cycles of learning and adaptation. We define the long-term interrelated development of human and computational intelligence across environment, actions, goal setting, and meaning extraction as *augmented intelligence (AI)*.

We present a detailed example of applying this methodology to the automation and augmentation of physical therapy assessment. We worked with 10 clinicians and 105 patients on the project for more than a year. We revealed explicit key components of the clinicians' embodied assessment process and modeled them as a Dynamic Bayesian Network (DBN). The DBN informed the design of a computational ensemble that successfully captured and extracted core elements of patient interactions with the environment and the therapist's assessment of those interactions. We could develop automated assessments that explicitly correlate clinically validated ratings with detailed movement-quality features. Clinicians believed that the enhancements provided by computational insights would help them improve their therapeutic strategies in ways that could be communicated explicitly to patients and other clinicians. Finally, we conclude by outlining key limitations and scaling opportunities for the methodology and discussing future study.

## Background and prior work

Interactions between humans and physical environments, as well as with other people, in tasks such as tying a shoelace (Korteling et al., 2021), performing surgery (Cooper and Tisdell, 2020; Kelly et al., 2019), or playing the violin in an orchestra (Proverbio and Valtolina, 2025; Clayton and Leante, 2013) require complex cognitive processes that are deeply intertwined with sensorimotor activities. The development of integrated cognitive and sensorimotor abilities through interactions with the environment enabled us to perform survival functions early in human development (hunting, food cultivation, building shelter, and making tools) and continued to evolve as our goals became more complex (the development of built environments and machines) (Varela et al., 2017). By creating artifacts that influenced our environments, we, in turn, influenced and evolved our interaction techniques (Dourish, 2001; Osiurak et al., 2018). For example, our current auditory scene analysis processes have their origins in human interactions with natural environments, but they have evolved to also address interactions with built environments (McAdams and Bigand, 1993).

Cognition is involved in the direct performance of human interactions with the environment and in the construction and manipulation of symbolic representations of these interactions (Simon, 1990). Symbolic representations facilitate the structured analysis of the components of our experience (Newell et al., 1989) and support the formation of symbolic logic (Pylyshyn, 1988). However, mental representations of an experience, like every form of modeling, rely on simplifying the experience to make it comprehensible, manipulable, and generalizable (Miller and Page, 2009). Furthermore, detailed and controlled analysis of an experience is typically achieved by isolating its components to avoid the complexities arising from their continuous interactions (Krakauer, 2024). This isolation removes much of the essence of that experience (Dewey, 2024). The proliferation of isolated analyses of components of human experience in academia can be partly attributed to the siloed approach of our learning and discovery institutions and structures (Darbellay, 2015) and to human “Descartian Anxiety” for analytic control (Varela et al., 2017). However, the more significant underlying reason may be that the development of symbolic logic and human rational thought is, overall, a more recent component of human evolution and thus comparatively limited (Ackerman, 1991; Korteling et al., 2021). Analysis of isolated components of an experience and the reduction of complexity through symbolic representations are well-suited to the limits of disembodied rational thought (Hutchins, 1995). Conversely, integrated cognitive and sensorimotor abilities for acting in the wild are much older in terms of human evolutionary time and are therefore more suited to engaging the totality of a complete experience (Moravec, 1988; Piaget, 1978; Maturana and Varela, 2012; Barsalou et al., 2003).

Conflating overall human cognition with disembodied symbol processing creates two key limitations. It marginalizes the most complex forms of human intelligence, which are embodied in action in the wild (Iida and Giardina, 2023; Hutchins, 1995; Johnson, 2024). It conflates human and computational intelligence, thereby reducing opportunities to design “non anthropomorphic artificial intelligence” (Korteling et al., 2021) that differs from but can synergize with embodied human intelligence (Monteith et al., 2024; Dave et al., 2023). Symbol-processing-based intelligence has the greatest potential to enrich human experience when integrated into the complex, embodied human experience. This integration can lead to reflection (Dewey, 2024; Varela et al., 2017), enhancing human experience and advancing human potential.

For an example of this integration, we can look to musical notation. Western classical music notation is about 1,000 years old (Burkholder et al., 2006). By the classical and romantic eras (1,700–1,900s), symbols (musical notations) were used to represent notes, dynamics (volume), and articulation in long musical pieces such as symphonies (>30 min in duration). The intelligent and predictable manipulation of symbols by expert musicians in an orchestra (approximately 100 people) could inform the coherent performance of millions of coordinated embodied actions by the musicians for over 30 min, producing a sonic experience that directly engaged and enriched 1,000 or more people in a large concert hall. David Krakauer described this type of integration of symbol processing into a complex experience as an example of “strong emergence in a causal function” that involves “low information source to high information targets” (Krakauer, 2024). There are two obvious cases of this enhanced information flow in the “above music example: the musicians augment the symbolic information given in the musical score in a predictable manner,” and the audience members augment the sonic information received from the symphony orchestra in a predictable manner (Krakauer, 2024). A third inverse information flow is involved in this complex experience: the composer needs to translate their complex experiential intentions into symbols (musical notation) so that the musicians can successfully augment these symbols into sounds that the audience can interpret as a complex and meaningful experience (Dewey, 2024; Burkholder et al., 2006).

The main reason symbols work so well in this type of emergence is that they successfully leverage highly developed forms of embodied human intelligence (Clayton and Leante, 2013). Musical notation and music experiences overall leverage thousandss of years of integrated development of sensorimotor activities and embodied cognition through interaction with the environment and society (Johnson, 2024; Barsalou et al., 2003). This shared embodied intelligence enables each performer to use a small set of common symbols to inform millions of sensorimotor activities that produce the desired sounds and to coordinate those activities with those of the other performers in real-time (Proverbio and Valtolina, 2025). It also allows the composer, the performers, and the audience to receive and interpret complex sequences of organized sound (including harmonic structure, melodic form, rhythmic organization, orchestration, and timbre) in a coherent and predictable manner (McAdams and Bigand, 1993; Clayton and Leante, 2013). But even more importantly, it is the combination of shared and individualized embodied intelligence that allows each audience member to receive and interpret the sounds of a symphony in a predictable manner and map that sonic experience to their own lived experience in a customized manner so they each feel the full strength of the experience (Dewey, 2024).

An art experience is a complex interplay among the expert practice of art-making, the produced artifact, the personal embodied experience of each audience member, and the shared embodied and cultural experiences of a society (Sloboda, 1986). As Vygotsky points out, this complexity surpasses the capacities of analytic thought and gives rise to aesthetics: an ancient Greek word for emotions and meaning arising from integrated multi-sensory perception and cognition (Vygotsky and Cole, 1978). During a recorded conversation with Maximilian Schell in 1972, Bernstein (1975) discusses the beginning of the second movement of Beethoven's Seventh Symphony. He explains that the “magic” of that moment cannot be in the music itself (the notes written on the page) because the musical material at that point is primarily a repeated note. The emergence of what Bernstein calls “magic” is due to the amplification of the simple musical artifact by the embodied experience of the listeners. This emergence would not be possible without the development of intricate and effective symbolic representation (musical notation). However, symbolic representation is neither the reason nor the substance of the experience. The synergy of symbolic representation and multifaceted embodied human intelligence produces a complex and powerful experience that is beyond direct analysis; it is emergent.

We propose that AI can integrate into the embodied human experience a level of symbol processing that has not been possible to date and that exceeds the capabilities of human symbol processing. The goal of this integration would not be to expand disembodied analysis. Instead, it aims to enable the emergence of augmented intelligence, in which human embodied intelligence, computational tools, and lived environments evolve in an integrative manner through complex interplay (Dave et al., 2023; Ehrenfeld, 2022). In the following section, we outline a methodology to advance the integration of AI into the performance of complex, embodied tasks.

## Methodology

### Representing performance of a complex task as a nested network

To facilitate human-AI collaboration, we focus this methodology on complex embodied tasks that involve multiple interdependent interactions among different people, tools, and environments, integrating predictability with uncertainty. The targeted tasks involve significant simultaneity of interactions at any given instance, along with a latent or explicit hierarchical time structure: instances are compiled into short and meaningful actions; sequences of actions are compiled into a defined performance of a complex task; and sequences of complex task performances enable iterative learning and improvement of performance (Iida and Giardina, 2023; Simon, 2019). The performance of these complex tasks requires specialized forms of embodied intelligence that are correlated with the extended practice of the task (Peña, 2010; Benner, 2004). The long-term collective experience of a community of expert practitioners (Sch'´on, 2017) yields partially formalized performances and assessments that can be tracked. The observable and formalized structures of these complex tasks provide an entry point for modeling and computation, while their latent components offer opportunities for improved performance through human-AI collaboration.

Human-AI collaboration requires an interaction model that is accessible to (and acceptable by) expert performers while also being computable. We propose building this interaction model on a nested network representation of the performance of the targeted complex task. The environment and human activities involved in a complex task include many features visible to both human and computational intelligence, and can thus support human-AI collaboration. Therefore, Environment (E) and Activity (A) form the two external layers of the nested network model. Within complex tasks, the environment and human activity are mutually defined (Varela et al., 2017; Osiurak et al., 2018). The elements of the environment shape the development of integrated human sensorimotor and cognitive strategies for effective performance within it; in turn, the human actor(s) influence the environment to improve performance. In many cases, these mutually defining interactions result in distributed cognition, in which the human actors design work environments, tools, and communication protocols to distribute processing across these components and improve performance (Hutchins, 1995; Dourish, 2001). Piloting large ships and planes and performing surgeries are complex tasks that exhibit the characteristics of distributed cognition (Cooper and Tisdell, 2020; Sergeeva et al., 2020; Hollan et al., 2000). Many of the tools and interactions of distributed cognition are gradually formalized and communicated through structured collections of symbols (i.e., the ship's navigation manual and navigation maps). The formalized interactions of distributed cognition (and the symbols that accompany them) provide key material that can be computationally captured and analyzed to support human-AI collaboration.

The third area of the nested network comprises the actors' goals (G), their embodied needs, and their motivations. Every complex task involves explicit goals and constituent sub-goals. For most formalized complex tasks, training manuals, textbooks, and other aids document the overarching goals and sub-goals of the task, linking these goals to the specific actions that human experts must take in particular situations and environments. For example, the main goals of an airline pilot are to “ensure they reach their destinations safely and securely” (ALPA, 2025). Sub-goals include: “work out the best route using weather reports and air traffic control data, create a flight plan, carry out pre-flight checks, follow procedures for take-off and landing, fly the plane, communicate with air traffic control, communicate with cabin crew and passengers, check data during the flight, and adjust the route if necessary, write reports” (National Careers Service, 2025). There are detailed pilot training manuals and flight manuals that connect these Goals (G) to Actions (A) that need to be taken by the pilot in the context of the immediate Environment (cockpit) and surrounding Environments (e.g., airplane, airports, flight paths) (FAA, 2025). Explicit goal documents and their connections of goals to actions and to structured environments can serve as the core starting point for a computable nested network model that is also readily accessible to expert performers of the task.

However, each complex task also has implicit and tacit goals and sub-goals. In many cases, these are not visible, as this knowledge is gained and shared by experts through their embodied collective practice (Sch'´on, 2017; Koshy et al., 2017; Benner, 2004). Each person's practice is partly driven by individualized needs and motivations. For example, it is critical for lawyers to understand diverse client needs and to present cases differently to different juries and judges. Although these goals and sub-goals are acknowledged in legal education, their achievement is primarily tacit and realized through collective and individualized reflective practice (Casey, 2013). The explicit goal of architects and clients is to design a building. However, different architects and clients may have varying motivations and needs regarding their functional and aesthetic choices (Sch'´on, 2017). Multi-actor complex tasks may involve actors with varied sub-goals and related expertise. For example, a surgical team comprises surgeons, anesthesiologists, nurses, and trainees, with distinct roles that must be coordinated to ensure successful surgical performance (Kelly et al., 2019). A human-AI collaboration model for a complex task needs to account for explicit goals and sub-goals, as well as hidden sub-goals, needs, and motivations of different actors (Prudkov, 2025; Granato and Baldassarre, 2024).

The fourth and innermost layer of the nested network is meaning (M). Since we are dealing with complex and embodied tasks, we use a definition of meaning that is based on related work from pragmatism and phenomenology (Dewey, 2024; Merleau-Ponty et al., 2013). The actor constructs meaning by connecting the different pieces of the integrated sensorimotor and cognitive experience they have while performing the complex task. The pieces that need to be connected for meaning extraction span the Environment, Activity, and Goals layers of our representation and establish multifaceted four-layer connectivity (across the EAGM layers) that is critical to the performance of the task (Fooken et al., 2023). The goal of extracting meaning is continuous improvement in the task, but also generalized learning that enhances the lived experience (Buschman and Miller, 2014; Tenenbaum et al., 2011; Granato and Baldassarre, 2024). Meaning extraction from complex embodied tasks is primarily achieved through reflection-in-action, but also through reflection-on-action (Varela et al., 2017; Dewey, 2024).

The professional development of nurses through training and practice is strongly associated with an increased ability to extract Meaning from complex interactions among Goals, Actions, and the Environment. When a nurse starts her professional career, she will primarily rely on the formal and explicit connections between Goals, Actions, and the Environment defined in the textbooks of her training (Kim, 1999; Peña, 2010). For example, “to determine fluid balance, nursing students are given clear parameters and guidelines: Check the patient's morning weights and daily intake and output for the past three days. Weight gain and intake consistently greater than 500 cc could indicate water retention, in which case fluid restriction should be started until the cause of the imbalance can be found” (Benner, 2004). However, fluid balance decisions are more salient and cannot be fully addressed by such rule-based interactions. As the nurse gains expertise, she must learn to guide her fluid balance decisions by considering a broader range of signs and symptoms (e.g., lethargy, skin turgor, mental status) as well as her interactions with the patient (Benner, 2004). She must also learn to address potential contradictory signals that may arise from the increased number of parameters and the competing needs and instructions from different stakeholders, including attending physicians, the patient, the patient's family, the hospital administration, and insurance (Mantzoukas and Jasper, 2004). Meaning extraction from complex interactions that involve a large number of Environment-Action-Goal parameters can only be achieved when these interactions have been “seen, recognized, and assessed across a range of patients” in actual practice (Benner, 2004).

The diagnoses, related treatment decision notes, and codes entered by the nurse in the EHR will provide an explicit symbolic representation of the Meaning the nurse extracted from a situation, as well as explicit connections of that Meaning to the environment (patient signs and symptoms), Activities (actions the nurse and/or other medical personnel have taken), and the Goals she was trying to achieve through her decision. The nurse's notes, along with other elements of the EHR, constitute the explicit, observable record of the patient's treatment by clinicians. However, many of the salient elements considered in her decision, and the reasons they informed it, may not appear in the EHR. An expert can intuitively explore many potential links between the components of complex task performance and, in many cases, produce new and highly impactful links that are not easily documented through traditional methods (Taylor, 1992). Bennet provides a detailed example of how an expert nurse deals with a situation where “a patient who was hemorrhaging stopped breathing. The links between the patient's condition and actions are sufficiently strong that the nurse attends primarily to actions rather than the assessment of signs and symptoms. This is reasonable because, in extreme circumstances, the possible responses are fewer, but experience is required to make this shift in performance” (Benner, 2004; Kim, 1999).

The tacit knowledge of experts is critical for extracting meaning from complexity and managing adverse (unexpected) events that cannot be captured or recorded in generalizable manuals across many types of complex tasks. The opening chapter of Edwin Hutchins's book “Cognition in the Wild” describes in detail an adverse event in which a large ship loses steam power (and eventually electricity) as it approaches the port (Hutchins, 1995). The experts in the pilothouse must quickly engage backup systems and emergency procedures that they rarely use, and establish new navigation goals, as the ship cannot enter port in its current state. They take actions (like different turns of the rudder) that test the behavior of the environment (the backup guidance mechanisms of the ship and the resulting overall direction of the ship) and use these interactions to extract meaning (calculate how long it would take for the ship to slow down without being able to reverse the engines and how well the ship could be guided with backup systems) and set new immediate goals (define a feasible location where the ship will have slowed down enough to drop anchor). As Hutchins puts it: “the safe arrival of the Palau (ship) at anchor was due in large part to the exceptional seamanship of the bridge crew” (Hutchins, 1995).

Performance of complex tasks by experts involves a combination of “explicit” knowing-that and “tacit” knowing-how (Peña, 2010; Polanyi, 2012). The augmented intelligence architecture we propose in this study leverages the symbolic and explicit connections among Meaning, Goals, Activity, and Environment to gradually reveal the tacit components and compute their dynamic connectivity with the explicit components, thereby increasing the observability of complex tasks performance. This increased observability enables the identification of areas of opportunity where AI can enhance human performance and informs the assessment of that performance. To facilitate this type of human-AI collaboration, the representation of human performance on complex tasks needs to seamlessly integrate the computational enhancements required for capture, processing, and interaction as an integral part of the performance.

**Figure 1:**
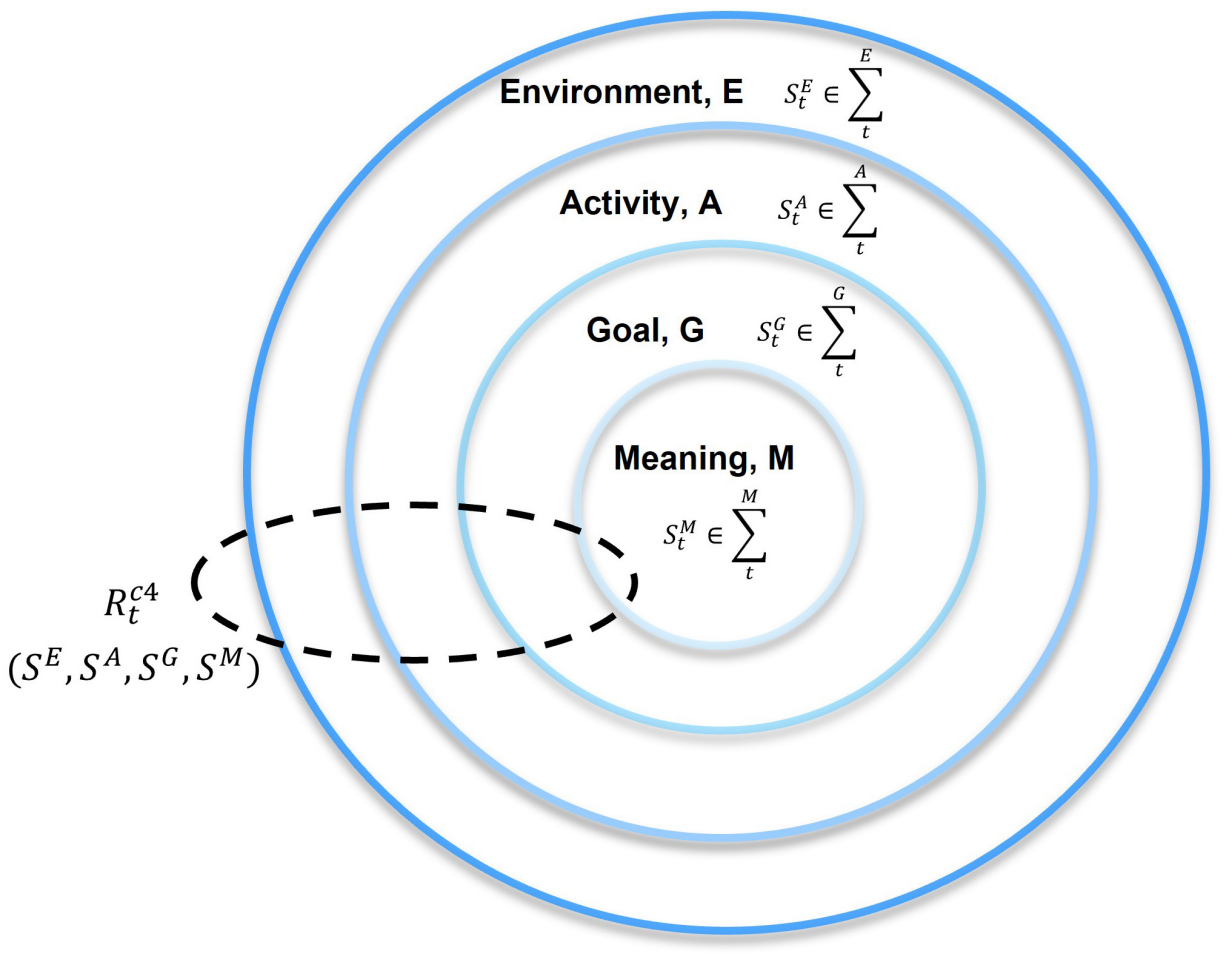
The nested network representing the performance of a complex task.

We propose that explainable and effective AI enhancements to human performance in complex tasks should be embedded in a four-layer nested network representation (Figure 1) that is computable. The assessment of the enhancement needs to be evident in the computational model and validated by human experts. Our nested network representation of performance on complex tasks shows all four constituent areas of the network as fully nested, indicating strong interdependence among its components. Changes to any component in any of the four areas of the network, whether through AI-based enhancements or other processes, will affect all areas and alter the network's configuration.

As discussed in the previous section, human performance on a complex task is clearly influenced by generalized learning that occurs outside the environment of the complex task. However, generalized learning connectivity is open and difficult to model in the context of specialized human-AI collaboration relating to the performance of a specific complex task (Tenenbaum et al., 2011). By modeling only the performance of a specialized, complex task, we do not need to represent all learning. Even in this limited case of a complex task, the network representation is dynamic and complex. We have argued that learning a complex, embodied task requires connectivity across all four layers of the network. Connectivity changes throughout the task as human actors and environments co-evolve. In a complex task, actors simultaneously manage a large number of nodes with multifaceted, dynamic connectivity. Almost all complex tasks must deal with unforeseen rare events (Krakauer, 2024).

Human actors manage this complexity in real time by distributing processing across the entire network and by relying on priors shaped by their embodied practice, as well as on the shared, accumulated knowledge of experts who perform and gradually formalize the complex task. Priors shaped by previous experience can be expressed as top-down conditional probabilities that, at any given point, may be affirmed or altered by the bottom-up data being observed (Fooken et al., 2023; Buschman and Miller, 2014; Tenenbaum et al., 2011; Spreng et al., 2010). We thus propose modeling the nested network representation as a bidirectional Dynamic Bayesian Network (DBN). In the next section, we discuss how the DBN can connect human expert performance to a computational ensemble, thereby informing system design and enhancing task performance.

### Co-designing the computational Ensemble for human-AI collaboration

The DBN supports system designers and expert task performers in the iterative co-design and implementation of human-AI collaboration for enhanced task performance. The DBN (i) *translates* the expert's symbols and routines (recorded in the nested network representation) into top–down information needs and bottom–up probabilistic updates; (ii) *informs* the design of digital tools (from sensors to data structures and AI engines) that can enhance task performance; (iii) *produces* symbolic descriptors and related calibrated likelihoods that increase the coherence and observability of the EAGM network, enhancing the probability of improved performance; and (iv) *assesses* enhanced human performance and computational design in an integrative manner.

#### Co-design of the nested network representation

The first step in the co-design process is to establish trust with experts in complex task performance by emphasizing the collaborative nature of the project. The AI will not attempt to replace the experts. Instead, it will be co-designed by AI developers and task experts to complement and enhance human embodied performance. The co-designers can then work together to define all explicit and observable components of the four layers of the nested network representation—*E*, *A*, *G*, *M*—and their explicit or potential connectivity. We restrict the model to *observable and explicitly defined* features at every step. This allows us to compute the E—A—G—M relationships of the nested network (Figure 1) directly in the probability space and to computationally track how AI-based enhancements improve the confidence of the system.

The co-design team should also define an explicit hierarchy of time periods for aggregating information in the nested network: (i) **instants** *t*; (ii) **actions** *k* (contiguous groups of instances that constitute a short meaningful action); (iii) a **performance** *T* (an ordered sequence of actions constituting one complex task performance); and (iv) a **sequence** ρ (a sequence of complex–task performances) enabling iterative learning across repeated performances.

#### Translating the observable descriptors and features into computable symbols

Once an initial network representation has been developed, the next step is to establish methods for capturing and annotating the expert performance of the task in a manner that preserves all features of the network representation in as much detail as possible, without interfering with the performance process. The AI designers need to build or select feature extractors that use the captured data to produce explicit mappings from expert descriptors to computable symbols. For example, the expert-defined boundary of an action can be coded as a specific interaction of recorded data fields so that the computer can look for those boundaries automatically. The raw captured streams should also be processed using appropriate AI algorithms (e.g., Transformers/LSTM for time-series analysis and CNNs for image-based analysis) to identify prominent patterns that may be tacitly used by experts but have not been explicitly defined.

#### Expression of the bidirectional DBN

The vocabulary of symbols created from the outputs of the feature extractors and expert descriptors populates the observation channels ${\text{Obs}}_{t}^{E},{\text{Obs}}_{t}^{A},{\text{Obs}}_{t}^{G},{\text{Obs}}_{t}^{M}$ of the DBN. At each instant *t* we map explicit and observable streams to finite vocabularies by symbolizers σ_•_:


$$\begin{array}{l} S_{t}^{E}=\sigma_{E}\left({\text{Obs}}_{t}^{E}\right)\in \Sigma_{t}^{E},\;S_{t}^{A}=\sigma_{A}\left({\text{Obs}}_{t}^{A}\right)\in \Sigma_{t}^{A},\\ S_{t}^{G}=\sigma_{G}\left({\text{Obs}}_{t}^{G}\right)\in \Sigma_{t}^{G},\;S_{t}^{M}=\sigma_{M}\left({\text{Obs}}_{t}^{M}\right)\in \Sigma_{t}^{M}. \end{array}$$ (1)

Here ${\text{Obs}}_{t}^{•}$ are observable features or descriptors; σ_•_ may be learned, or rule-based; and $\Sigma_{t}^{•}$ (the lexicons) may evolve over time as computational enhancements propose candidates for inclusion that can be accepted or rejected based on the bidirectional DBN processes formalized in the later sections.

**Figure 2:**
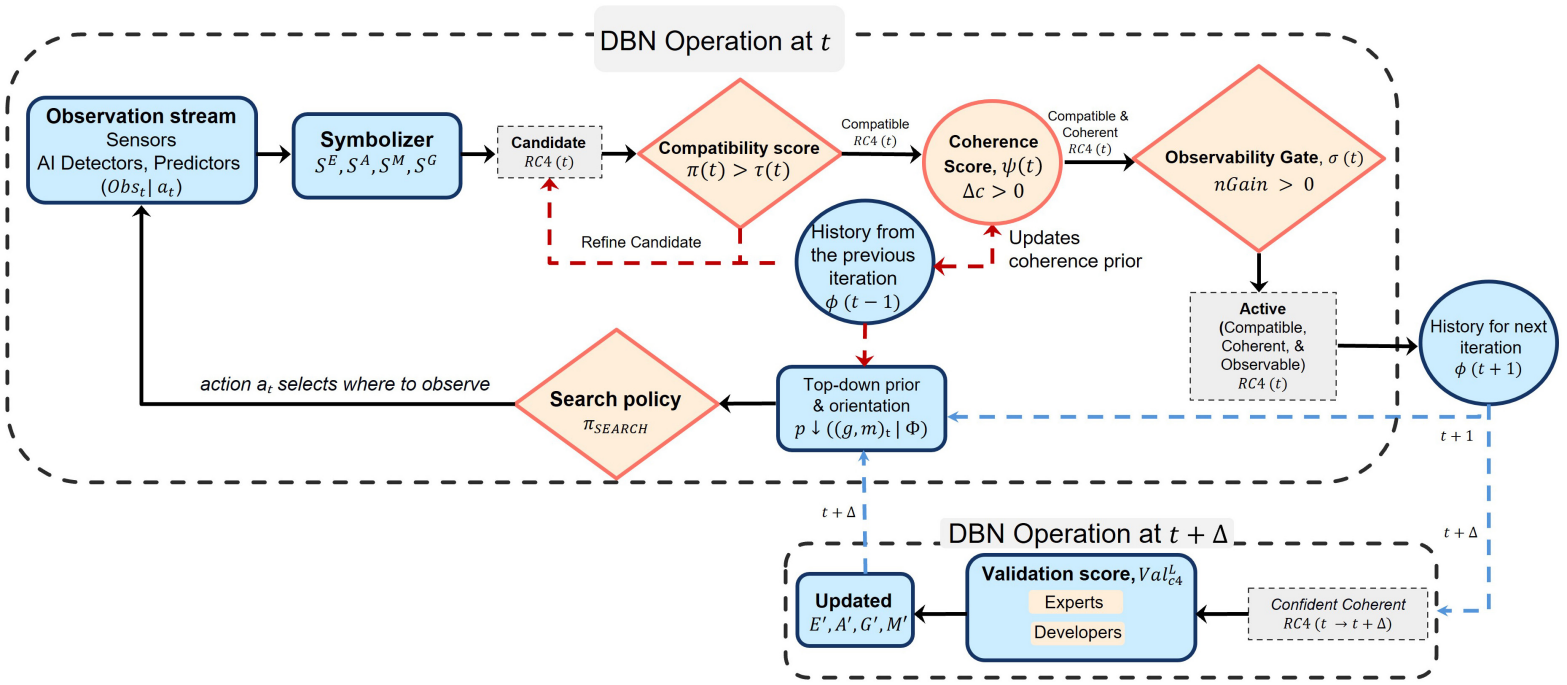
Bidirectional DBN operation showing the complete processing cycle. DBN Operation at *t* (dashed boundary): The top-down pass begins with history Φ(*t*−1) parameterizing the prior over orientation (*g, m*)_*t*_, which drives the search policy π_SEARCH_ to select action *a*_*t*_. The bottom-up pass processes *Obs*(*t*∣*a*_*t*_) through symbolizers to produce candidate quadruples $R^{c4}\left(t\right)$. Candidates pass sequentially through: (1) compatibility check κ(*t*) > τ(*t*), (2) coherence gate ψ(*t*) requiring ΔC>0, and (3) observability gate σ(*t*) requiring *nGain* > 0. Candidates failing any gate (dashed red paths) are refined and re-evaluated; persistent failures update the error list and prior. Candidates passing all gates become Active $R^{c4}\left(t\right)$—compatible, coherent, and observable—and feed into history Φ(*t*+1) for the next iteration. DBN Operation at *t*+Δ: After sufficient iterations, when active candidates have accumulated confidence, they proceed to the validation gate where experts and developers confirm integration. Only validated candidates update the EAGM network (*E*′, *A*′, *G*′, *M*′), producing confident coherent $R^{c4}\left(t\to t+\Delta \right)$ that becomes part of the established priors.

Before presenting the detailed mathematical formulation of the DBN, we provided a high-level view of the bidirectional inference cycle illustrated in Figure 2. A single DBN step contains a **top-down pass** (predict orientation, choose where to look) and a **bottom-up pass** (observe and symbolize, build cross-layer connectors, update orientation, and gate candidate codes). Combining both passes yields a joint posterior over orientation, symbols, and connecting edges across all four layers of the network. Because the four layers (EAGM) are nested, the bottom-up pass must first decide, at each instant, *which four layer combinations* (*s*^*E*^, *s*^*A*^, *s*^*G*^, *s*^*M*^) *are actually in effect*, and the top-down pass must read a compact *summary of what has been happening* across time brackets (actions, tasks, and sequences) to set expectations. Accordingly, we (i) defined how a candidate quadruple becomes an active *c*4 edge at an instant, and (ii) defined how those instantaneous edges are aggregated into fixed-size summaries that seed the top-down prior and the discovery gates.

The DBN operates through these complementary passes within a gated pipeline that determines which cross-layer quadruples (*s*^*E*^, *s*^*A*^, *s*^*G*^, *s*^*M*^) ultimately become active edges (*c*4) and, after sufficient confidence accumulates, are part of the updated nested network. The *top-down pass* begins with the accumulated history Φ(*t*−1) from previous instants, actions, and task performances. This history parameterizes a prior distribution over the current orientation (*g, m*)_*t*_—encoding “what goal (g) are we pursuing” and “how do we currently interpret the unfolding performance (meaning-m).” The orientation drives a search policy π_SEARCH_ that selects an action *a*_*t*_ to maximize expected information gain. This action conditions the observation that initiates the bottom-up pass.

The *bottom-up pass* processes the action-conditioned observation *Obs*(*t*∣*a*_*t*_) through layer-specific symbolizers, yielding candidate symbols $\left(S_{t}^{E},S_{t}^{A},S_{t}^{G},S_{t}^{M}\right)$. These symbols are assembled into candidate quadruples *r* = (*s*^*E*^, *s*^*A*^, *s*^*G*^, *s*^*M*^), collectively denoted $R^{c4}\left(t\right)$. Each candidate must then pass through a sequence of three gates—compatibility, coherence, and observability—before becoming an active edge. Candidates that fail any gate are routed back for refinement; those that fail repeatedly are moved to an error list that feeds back to update the prior. The bottom-up pass concludes with the *orientation update*: fusing the top-down prior with the bottom-up evidence (active symbols and *c*4 edges) to obtain a joint posterior over orientation. This posterior becomes the starting point for the next top-down pass.

Active $R^{c4}\left(t\right)$ edges flow into the history for the next iteration Φ(*t*+1), but they do not immediately update the EAGM network. Instead, they accumulate confidence over a period Δ. Only after sufficient iterations—when the active candidates have gained confidence above a threshold—do they proceed to the *validation gate*, where experts and developers confirm the integration of the new patterns. Upon validation, the network state (*E*′, *A*′, *G*′, *M*′) is finally updated, closing the outer loop shown in Figure 2.

We now formalize each component of this cycle.

##### Top-down pass: orientation prior and search policy

The latent *orientation* (*g*_*t*_, *m*_*t*_) summarizes “what we are trying to do” (*g*_*t*_: current goal) and “how we currently interpret the unfolding performance” (*m*_*t*_: meaning). This orientation is drawn from a top-down prior:


$$(g_{t},m_{t})\sim p_{↓}\text{​}({\left(g,m\right)}_{t}|{\left(g,m\right)}_{t-1},\;\Phi_{\leq t-1},\;\Lambda_{t}),$$ (2)

where $\Lambda_{t}=\left(\Sigma_{t}^{E},\Sigma_{t}^{A},\Sigma_{t}^{G},\Sigma_{t}^{M},\;\sigma_{E},\sigma_{A},\sigma_{G},\sigma_{M},\;\kappa_{t}\right)$ encodes the current lexicon state. The conditional is a **top-down prior**—it is computed *before* looking at the current evidence at time *t*. Hence, it conditions on the previous orientation (*g, m*)_*t*−1_, fixed-size summaries Φ_≤ *t*−1_ from earlier aggregates (actions, tasks, sequences), and the current lexicon state Λ_*t*_.

The predicted orientation guides *where to look next*: if certain goals or interpretations are the most uncertain or the most consequential now, the policy selects actions that are expected to be diagnostic for them. This corresponds to the “Search policy π_SEARCH_” defined as:


$$a_{t}\sim \pi_{\text{SEARCH}}\text{​}(a|g_{t},m_{t},\;S_{t-1}^{E},S_{t-1}^{A},\;\Lambda_{t}),$$ (3)

which maximizes expected information gain minus action cost:


$$a_{t}\in \text{arg }\underset{a\in A}{\text{max}}\underset{\text{reduce uncertainty in }(g,m)}{\underset{︸}{E[{\text{IG}}_{t}(a)]}}\;-\text{ cost}(a).$$ (4)

The selected action *a*_*t*_ conditions the observation stream, yielding *Obs*(*t*∣*a*_*t*_).

##### Action cost: constraining search to expected connectivity

We formalize the action cost to reflect a key insight: given the current orientation (*g*_*t*_, *m*_*t*_) derived from history Φ_≤ *t*−1_, the DBN has well-defined expectations for which Environment–Activity combinations are relevant. Searching outside this constrained space increases both time and uncertainty.

Let $A_{exp}\left(g_{t},m_{t}\right)\subseteq A$ denote the *expected action set*—the computational actions (e.g., sensor orientation, sensor combinations, feature extraction, and computation of feature correlations) that are likely to reveal evidence consistent with the current orientation. This set is derived from the history: given (*g, m*)_*t*−1_ and the coherence prior $\pi_{t}^{\text{coh}}$, the DBN knows which *E*–*A* combinations have historically co-occurred with and/or right after the current goal–meaning state.

So, the action cost decomposes into two terms:


$$\text{cost}(a_{t})=\underset{\text{execution time}}{\underset{︸}{c_{\tau }\cdot \tau (a_{t})}}+\underset{\text{deviation from expectations}}{\underset{︸}{c_{\text{dev}}\cdot d(a_{t},A_{\text{exp}}(g_{t},m_{t}))}}.$$ (5)

*Execution time* τ(*a*_*t*_) captures the resource cost of performing action *a*_*t*_—switching sensors, extracting more features, and calculating new feature correlations all consume time and computational resources. The coefficient *c*_τ_ > 0 weights this base cost.

*Deviation from expectations* measures how far *a*_*t*_ lies from the expected action set. We define:


$$d(a_{t},A_{\text{exp}}(g_{t},m_{t}))=-\log\pi_{\text{exp}}(a_{t}|g_{t},m_{t},\Phi_{\leq t-1}),$$ (6)

where π_*exp*_(*a*∣*g, m*, Φ) is the probability that action *a* is aligned with the expected *E*–*A* connectivity given orientation (*g, m*) and history Φ. This probability is derived from the coherence prior:


$$\pi_{\text{exp}}(a_{t}|g_{t},m_{t},\Phi_{\leq t-1})=\sum_{r:a_{t}\in \text{supp}(r)}\pi_{t}^{\text{coh}}(r)\cdot p(g_{t},m_{t}|r),$$ (7)

where the sum is over quadruples *r* = (*s*^*E*^, *s*^*A*^, *s*^*G*^, *s*^*M*^) whose *E*–*A* components are accessible via action *a*_*t*_. Actions that search within the expected connectivity have high π_*exp*_ and thus low deviation costs; actions that search randomly have low π_*exp*_ and incur high costs.

The scalar coefficients (*c*_τ_, *c*_*dev*_) are learned from the data, as their optimal values depend on the specific task and the reliability of the coherence prior. Early in co-design (sparse history), *c*_*dev*_ should be low to encourage exploration; as the prior stabilizes, *c*_*dev*_ increases to exploit accumulated knowledge. This adaptive weighting ensures that the system balances exploration and exploitation as it learns.

Substituting Equation 5 into the search objective (Equation 4), the optimal action is:


$$a_{t}=\text{arg }\underset{a\in A}{\text{max}}\{E[{\text{IG}}_{t}(a)]-c_{\tau }\tau (a)-c_{\text{dev}}\cdot d(a,A_{\text{exp}})\}.$$ (8)

This formulation ensures that the search policy prioritizes actions within the expected connectivity space, deviating only when the expected information gain is much higher than anticipated—denoting the possibility of a novel or unknown event in task performance that justifies a momentary increase in time and deviation costs.

##### Bottom-up pass: action-conditioned symbolization and candidate discovery

The *bottom-up pass* processes the action-conditioned observation *Obs*(*t*∣*a*_*t*_) through layer-specific symbolizers, yielding candidate symbols $\left(S_{t}^{E},S_{t}^{A},S_{t}^{G},S_{t}^{M}\right)$. Critically, the observation at instant *t* depends on the action *a*_*t*_ selected by the search policy. Different actions yield different observations, and we make this dependency explicit in the symbolization equations. The action-conditioned observations ${\text{Obs}}_{t}^{L}\left(a_{t}\right)$ are converted to symbols:


$$\begin{array}{l} S_{t}^{E}\sim p(\cdot |{\text{Obs}}_{t}^{E}\left(a_{t}\right),\Sigma_{t}^{E}),\;S_{t}^{A}\sim p(\cdot |{\text{Obs}}_{t}^{A}\left(a_{t}\right),\Sigma_{t}^{A}),\\ S_{t}^{G}\sim p(\cdot |{\text{Obs}}_{t}^{G}\left(a_{t}\right),\Sigma_{t}^{G}),\;S_{t}^{M}\sim p(\cdot |{\text{Obs}}_{t}^{M}\left(a_{t}\right),\Sigma_{t}^{M}). \end{array}$$ (9)

Here ${\left\{p\left(\cdot |{\text{Obs}}_{t}^{L}\left(a_{t}\right),\Sigma_{t}^{L}\right)\right\}}_{L\in \left\{E,A,G,M\right\}}$ are calibrated observation factors that map action-conditioned observations—obtained from captured data by domain-specific extractors—into distributions over explicit symbols, with quantified uncertainty. The observation ${\text{Obs}}_{t}^{L}\left(a_{t}\right)$ reflects what is revealed when action *a*_*t*_ is taken: different actions expose different aspects of the Environment, Activity, Goals, and Meaning. Predictions from an ensemble of AI modules pertinent to the current state directly parameterize these terms.

When current vocabularies $\Sigma_{t}^{L}$ have low confidence in explaining the observed structure, the bottom-up pass also activates a *proposal mechanism* $p\left({\tilde{S}}_{t}^{L}|\cdot \right)$ that generates candidate explicit codes in layer *L*. Typical triggers include: (i) high residuals in the builder (many near-miss κ_*t*_ patterns), (ii) stable co-occurrence clusters of partial tuples (e.g., (*s*^*E*^, *s*^*A*^, *, *)), and (iii) expert-suggested codes from interactions. AI extractors and predictors help in two ways: by surfacing stable feature clusters as proposals and by improving calibration so that the gate's utility and coherence tests are reliable. For the discovery and updating of vocabularies:


$$\begin{array}{l} {\tilde{S}}_{t}^{L}\sim p\left(\cdot |H_{\leq t},\Lambda_{t}\right),\;u_{t}^{L}\left(s\right)\in \left\{0,1\right\},\\ \Sigma_{t+1}^{L}=\Sigma_{t}^{L}\cup \left\{s\in {\tilde{S}}_{t}^{L}:u_{t}^{L}\left(s\right)=1\right\},\;L\in \left\{E,A,G,M\right\}. \end{array}$$ (10)

The gate decision $u_{t}^{L}\left(s\right)$ determines whether a proposed symbol *s* is accepted into the vocabulary. With symbols in hand—whether from existing vocabularies or newly proposed candidates—we decide which quadruples *r* = (*s*^*E*^, *s*^*A*^, *s*^*G*^, *s*^*M*^) are candidate cross-layer edges at time *t*, producing the “Candidate $R^{c4}\left(t\right)$” shown in Figure 2.

##### Bottom-up pass: the *c*4 builder and gated pipeline

The *c*4 builder assembles candidate quadruples and determines which become active edges through a sequence of three gates. Because a *c*4 edge encodes a strong cross-layer constraint over *E*–*A*–*G*–*M*, calibrated AI predictors that assign high and mutually consistent probabilities to the symbols in *r* = (*s*^*E*^, *s*^*A*^, *s*^*G*^, *s*^*M*^) increase the compatibility potential κ_*t*_(*r*).

###### Gate 1 (Instantaneous edge activation): compatibility check

Consider a candidate quadruple *r* = (*s*^*E*^, *s*^*A*^, *s*^*G*^, *s*^*M*^). The compatibility score κ_*t*_(*r*) evaluates whether the proposed connectivity is compatible with existing connectivity at instant *t*. Compatibility enforces two fundamental rules:

Rule 1 (Novel elements compatible with existing connectivity): the candidate may propose *novel* connections for an existing EAGM connectivity pattern (linking elements not previously connected to the pattern); however, those novel connections cannot *contradict* the well-established Gs and Ms of the connectivity pattern. Thus, the additions of pertinent E and A elements to existing Gs and Ms are allowed, and the additions of pertinent sub-Goals and sub-Meanings are also permitted.

Rule 2 (Novel elements with novel four-layer connectivity): novel elements that do not meet Rule 1 can be suggested as long as they have novel four-layer connectivity (i.e., novel E and A elements with novel G and M connections), thus indicating a new or alternative interpretation of the current events of the task.

These rules ensure that compatibility acts as a filter for genuine novelty: candidates that shift multiple elements together in a way that extends (rather than opposes) the existing network structure pass through, while arbitrary single-element changes or contradictory proposals are rejected.

The probability that *r* passes the compatibility check and proceeds to the coherence gate is:


$$\begin{array}{l} p(r\text{ passes compatibility})=1\{\kappa_{t}\left(r\right)>\tau_{t}\}, \end{array}$$ (11)

where $\kappa_{t}:\Sigma_{t}^{E}\times \Sigma_{t}^{A}\times \Sigma_{t}^{G}\times \Sigma_{t}^{M}\to \left[0,1\right]$ is the compatibility score and τ_*t*_ ∈ (0, 1) is a threshold. To encourage exploration early and selectivity later within an action or performance, we adopt a monotonic schedule:


$$\tau_{t}=\tau_{\min}+\left(\tau_{\max}-\tau_{\min}\right)s\left(\pi_{t}\right),$$

where π_*t*_ ∈ [0, 1] denotes normalized progress within the current action and *s*:[0, 1] → [0, 1] is an increasing schedule (e.g., *s*(*x*) = *x*^*p*^, *p*≥1).

###### Gate 2 (Aggregated evidence over time): coherence check

Candidates passing the compatibility check proceed to the coherence gate. Unlike compatibility, which is an instantaneous assessment, coherence requires evidence aggregated over time. One cannot assess coherence at a single instant—coherence measures whether a pattern aligns with what has been observed across actions, tasks, and sequences.

After inferring the DBN posterior at time *t*−1, we compress beliefs into hierarchical summaries that inform the coherence assessment. Let *r* = (*s*^*E*^, *s*^*A*^, *s*^*G*^, *s*^*M*^) index a candidate *c*4 edge and $C_{t}^{inst}\left(r\right)=\text{1}\left\{r\in R_{t}^{c4}\right\}$ be the instant-level indicator. Using evidence up to *t*−1, we form prefix counts at each aggregate level:


$$\begin{array}{l} C_{k,\leq t-1}^{\text{act}}\left(r\right)=\underset{s\in I_{k}:\;s\leq t-1}{\sum }C_{s}^{\text{inst}}\left(r\right),\;N_{k,\leq t-1}^{\text{act}}=\underset{r}{\sum }C_{k,\leq t-1}^{\text{act}}\left(r\right), \end{array}$$ (12)

$$\begin{array}{l} C_{T,\leq t-1}^{\text{task}}\left(r\right)=\underset{k\in U_{T}}{\sum }C_{k,\leq t-1}^{\text{act}}\left(r\right),\;N_{T,\leq t-1}^{\text{task}}=\underset{r}{\sum }C_{T,\leq t-1}^{\text{task}}\left(r\right), \end{array}$$ (13)

$$\begin{array}{l} C_{\rho ,\leq t-1}^{\text{seq}}\left(r\right)=\underset{T\in T_{\rho }}{\sum }C_{T,\leq t-1}^{\text{task}}\left(r\right),\;N_{\rho ,\leq t-1}^{\text{seq}}=\underset{r}{\sum }C_{\rho ,\leq t-1}^{\text{seq}}\left(r\right). \end{array}$$ (14)

Converting counts to probabilities with Dirichlet smoothing:


$$\begin{array}{l} \pi_{k}^{\text{act}}\left(r\right)=\frac{C_{k,\leq t-1}^{\text{act}}\left(r\right)+\alpha b\left(r\right)}{N_{k,\leq t-1}^{\text{act}}+\alpha },\;\pi_{T}^{\text{task}}\left(r\right)=\frac{C_{T,\leq t-1}^{\text{task}}\left(r\right)+\alpha b\left(r\right)}{N_{T,\leq t-1}^{\text{task}}+\alpha },\\ \pi_{\rho }^{\text{seq}}\left(r\right)=\frac{C_{\rho ,\leq t-1}^{\text{seq}}\left(r\right)+\alpha b\left(r\right)}{N_{\rho ,\leq t-1}^{\text{seq}}+\alpha }. \end{array}$$ (15)

The coherence prior at instant *t* is the convex mixture:


$$\pi_{t}^{\text{coh}}\left(r\right)=w_{\text{act}}\pi_{k\left(t\right)}^{\text{act}}\left(r\right)+w_{\text{task}}\pi_{T\left(t\right)}^{\text{task}}\left(r\right)+w_{\text{seq}}\pi_{\rho \left(t\right)}^{\text{seq}}\left(r\right),\;\sum w_{\cdot }=1,$$

with log-odds $\psi_{t}^{\text{coh}}\left(r\right)=\log\frac{\pi_{t}^{\text{coh}}\left(r\right)}{1-\pi_{t}^{\text{coh}}\left(r\right)}$.

The coherence gate requires a positive coherence gain:


$$\begin{array}{l} \Delta C_{\text{agg}}^{L}\left(r\right)=\underset{X\in \left\{\text{act},\text{task},\text{seq}\right\}}{\sum }w_{X}(C_{X}^{\text{new}}-C_{X}^{\text{old}})>0, \end{array}$$ (16)

where $C_{X}$ measures alignment with historically frequent patterns at level *X*. Candidates with ΔC>0 pass the coherence gate and become part of the *coherent* $R^{c4}$ set.

###### Gate 3 (Demonstrating increased connectivity): observability check

Candidates passing the coherence gate proceed to the observability gate, which evaluates whether accepting the candidate increases the observable cross-layer connectivity of the network. We define the observability gain:


$$\begin{array}{l} {\text{nGain}}_{c4}^{L}\left(t\right)=\frac{\;E[|R_{t}^{c4}|\;|\;\Sigma_{t}^{L,\text{new}}]-E[|R_{t}^{c4}|\;|\;\Sigma_{t}^{L,old}]\;}{|\Sigma_{t}^{E}|\cdot |\Sigma_{t}^{A}|\cdot |\Sigma_{t}^{G}|\cdot |\Sigma_{t}^{M}|\;\vee \;1}. \end{array}$$ (17)

The numerator is the expected increase in active, compatible *c*4 edges because the vocabulary was augmented. The denominator normalizes by the ambient search space, rewarding expressivity that manifests rather than raw vocabulary growth.

Candidates with *nGain* > 0 pass the observability gate and become *active* $R^{c4}\left(t\right)$ edges—compatible, coherent, and observable.

###### Refinement loop and error handling

Candidates failing any of the three gates are not simply discarded. Instead, they enter a refinement loop (dashed red paths in Figure 2). The system attempts to adjust symbolizer parameters, recalibrate compatibility thresholds, seek additional observations, or modify the candidate by considering alternative symbol combinations.

Candidates cycle through refinement until they either pass all gates or exhaust a maximum number of attempts. Candidates who persistently fail are moved to an *error list*. Importantly, the error list is not a dead end—it feeds back to update the prior, ensuring that the system learns from failures. Patterns that consistently fail may indicate: (i) sensor or symbolizer miscalibration requiring engineering attention, (ii) genuine edge cases that experts should review, or (iii) noise that should be down-weighted in future assessments.

##### Bottom-up pass: orientation update via joint posterior

The bottom-up pass concludes by fusing the top-down prior with the bottom-up evidence to obtain a joint posterior over orientation, symbols, and *c*4 edges. Let $H_{t-1}$ denote the history up to *t*−1 (including the posterior over (*g, m*)_*t*−1_ and the summaries Φ_≤ *t*−1_). At instant *t*, the action *a*_*t*_ is chosen by the search policy (Equation 3), and we condition on this realized *a*_*t*_ when forming observations.

Define the per-layer symbolization likelihood (Equation 9) and the *c*4 builder likelihood:


$$\begin{array}{l} L_{t}\left(a_{t}\right)\equiv [\prod_{L\in \left\{E,A,G,M\right\}}p(S_{t}^{L}|{\text{Obs}}_{t}^{L}\left(a_{t}\right),\Sigma_{t}^{L})]\\ p(R_{t}^{c4}|S_{t}^{E},S_{t}^{A},S_{t}^{G},S_{t}^{M},\kappa_{t},\psi_{t}^{\text{coh}}). \end{array}$$ (18)

Using the top-down prior (Equation 2) and the per-layer likelihood $L_{t}\left(a_{t}\right)$, the step-*t* joint posterior over orientation, symbols, and *c*4 edges, given the realized action and observations, factorizes as:


$$\begin{array}{cc} p_{t}({\left(g,m\right)}_{t},S_{t}^{E},S_{t}^{A},S_{t}^{G},S_{t}^{M},R_{t}^{c4}|H_{t-1},a_{t},{\text{Obs}}_{t}\left(a_{t}\right)) & \propto \\ p_{↓}({\left(g,m\right)}_{t}|{\left(g,m\right)}_{t-1},\Phi_{\leq t-1},\Lambda_{t})\times L_{t}\left(a_{t}\right). \end{array}$$ (19)

The instantaneous log-posterior satisfies:


$$\begin{array}{r} \log p({\left(g,m\right)}_{t}|\text{history},a_{t})\propto \\ \underset{\text{top-down expectations}}{\underset{︸}{\log p_{↓}({\left(g,m\right)}_{t}|\cdot )}}+\underset{\text{action-conditioned bottom-up evidence}}{\underset{︸}{\log L_{t}\left(a_{t}\right)}}, \end{array}$$

Any improvements that sharpen the symbolization likelihoods or strengthen *c*4 consistency (higher κ_*t*_, more informative $\psi_{t}^{\text{coh}}$) tighten the posterior. This joint posterior represents the fusion of top-down and bottom-up passes at each instant.

We can define the prediction from the top-down prior by averaging over the previous posterior:


$$\begin{array}{r} {\tilde{p}}_{t}({\left(g,m\right)}_{t}):=E_{\left(g^{\prime },m^{\prime }\right)\sim p\left({\left(g,m\right)}_{t-1}|H_{t-1}\right)}\\ {}[p_{↓}({\left(g,m\right)}_{t}|\left(g^{\prime },m^{\prime }\right),\Phi_{\leq t-1},\Lambda_{t})]. \end{array}$$ (20)

Conditioned on the realized action *a*_*t*_, symbols, and *c*4 edges at time *t*, the *posterior kernel* over orientation is:


$$\begin{array}{r} p_{↑}({\left(g,m\right)}_{t}|S_{t}^{E},S_{t}^{A},S_{t}^{G},S_{t}^{M},R_{t}^{c4},a_{t},H_{t-1},\Lambda_{t})\propto \\ {\tilde{p}}_{t}({\left(g,m\right)}_{t})\;p(R_{t}^{c4}|S_{t}^{E},S_{t}^{A},S_{t}^{G},S_{t}^{M},\kappa_{t},\psi_{t}^{\text{coh}}). \end{array}$$ (21)

A point estimate for orientation (if needed) is then obtained as follows:


$$\begin{array}{l} \left({\hat{g}}_{t},{\hat{m}}_{t}\right)\in \arg\underset{\left(g,m\right)}{\max}\;p_{↑}({\left(g,m\right)}_{t}|S_{t}^{E},S_{t}^{A},S_{t}^{G},S_{t}^{M},R_{t}^{c4},a_{t},H_{t-1},\Lambda_{t}). \end{array}$$ (22)

This orientation update completes the bidirectional inference at instant *t*: the top-down prior set expectations, the bottom-up pass gathered evidence and built active *c*4 edges, and the joint posterior now reflects both. The updated orientation (*g*_*t*_, *m*_*t*_) becomes the starting point for the next top-down pass.

##### History update and feedback to instantaneous decisions

Active $R^{c4}\left(t\right)$ edges—having passed compatibility, coherence, and observability gates—flow into the history for the next iteration. Let $I_{k}$ denote the instants in action *k*, $U_{T}$ the actions in task *T*, and *T*_ρ_ the tasks in sequence ρ. If the instantaneous state is $H_{t}=\left(S_{t}^{E},S_{t}^{A},S_{t}^{G},S_{t}^{M},\;R_{t}^{c4}\right)$, the summaries are:


$$\begin{array}{r} H_{k}^{\text{act}}=\text{Agg}(\left\{H_{t}:\;t\in I_{k}\right\}),\\ H_{T}^{\text{task}}=\text{Compose}(\left\{H_{k}^{\text{act}}:\;k\in U_{T}\right\}),\\ H_{\rho }^{\text{seq}}=\text{Compose}(\left\{H_{T}^{\text{task}}:\;T\in T_{\rho }\right\}). \end{array}$$ (23)

Here, Agg pools sufficient statistics within an action (e.g., *c*4 histograms, dwell times, transitions), and Compose concatenates child summaries carrying cross-boundary information upward. The fixed-size summary:


$$\begin{array}{l} \Phi_{\leq t-1}\equiv \Phi (\left\{H_{k^{\prime }<k\left(t\right)}^{\text{act}}\right\},\;\left\{H_{T^{\prime }<T\left(t\right)}^{\text{task}}\right\},\;\left\{H_{\rho^{\prime }\leq \rho \left(t\right)}^{\text{seq}}\right\}) \end{array}$$ (24)

includes normalized edge histograms {π^*act*^, π^*task*^, π^*seq*^} used to compute the coherence prior for subsequent iterations.

This history feeds back to influence instantaneous decisions in two ways. First, at instant *t*, the mixture prior $\pi_{t}^{\text{coh}}\left(r\right)$ and its log-odds $\psi_{t}^{\text{coh}}\left(r\right)$ bias the selection of *E*–*A*–*G*–*M* edges toward patterns that have repeatedly co-occurred at the current *action* *k*(*t*), *task* *T*(*t*), and *sequence* ρ(*t*) levels. This feedback from history to the coherence prior is demonstrated in Figure 2.

Second, when a new candidate symbol $s_{\text{new}}^{L}$ or edge *r*_new_ *passes* all gates, it is promoted to the likelihood. These updates immediately change the next-step evidence:


$$\begin{array}{c} L_{t+1}\left(a_{t+1}\right)\\ =[\prod_{L}p\left(S_{t+1}^{L}|{\text{Obs}}_{t+1}^{L}\left(a_{t+1}\right),\Sigma_{t+1}^{L}\right)]\\ p(R_{t+1}^{c4}|S_{t+1}^{E},S_{t+1}^{A},S_{t+1}^{G},S_{t+1}^{M},\kappa_{t+1},\psi_{t+1}^{\text{coh}}), \end{array}$$

because (i) the symbolization likelihoods can now assign calibrated probability mass to the new code(s), and (ii) the coherence prior $\psi_{t+1}^{\text{coh}}$ increases for motifs involving *r*_new_ (as the aggregated counts rise). Consequently, the posterior $p\left({\left(g,m\right)}_{t+1}|H_{t+1},a_{t+1}\right)$ tightens around orientations consistent with the newly stabilized *E*–*A*–*G*–*M* structure. Candidates that *fail* the gates remain auxiliary (used for learning and calibration) and do not enter $L_{t}$, leaving the posterior unchanged by them. Crucially, active candidates update the history Φ(*t*) → Φ(*t*+1) but do *not* immediately update the EAGM network (*E*′, *A*′, *G*′, *M*′). The network update requires validation.

##### Validation and network update at *t*+Δ

Active $R^{c4}$ edges accumulate confidence through repeated activation across iterations. After a period Δ—which may span multiple actions or even tasks—candidates who have maintained active status with confidence above a threshold proceed to the *validation gate*.

At the validation gate, experts and developers review the accumulated evidence:

- **Developers** verify that the technical implementation (sensors, feature extractors, symbolizers, derivation algorithms) is correctly calibrated and that the observed patterns reflect genuine signals rather than artifacts.
- **Experts** confirm that the patterns have domain relevance—that they correspond to meaningful distinctions in complex task performance.

The validation score ${val}_{c4}^{L}$ captures this expert assessment. The complete gate for network update combines accumulated confidence with validation:


$$\begin{array}{l} \mathrm{Pr}[u_{t}^{L}\left(s\right)=1]=\sigma (\lambda_{\text{conf}}\;{\text{Conf}}_{c4}^{L}\left(s\right)+\lambda_{\text{val}}\;{\text{val}}_{c4}^{L}), \end{array}$$ (25)

where ${\text{Conf}}_{c4}^{L}\left(s\right)$ measures accumulated confidence from repeated activation.

Only candidates that pass this validation gate update the EAGM network (*E*′, *A*′, *G*′, *M*′). This collaboration ensures that: (i) instantaneous decisions respect existing knowledge while remaining open to genuine novelty; (ii) only candidates with demonstrated temporal coherence and observability enter the active set; (iii) experts are not burdened with instantaneous decisions but engage only after patterns have proven stable; (iv) the system now has a new posterior peak and lowest entropy; and (v) the system continuously learns from both successes and failures.

## Experiment and results: human AI collaboration in physical therapy assessment

Physical therapy for stroke survivors is a complex task requiring interdependent interactions between therapists and stroke survivors, as well as standardized tools and environments. Therapy may last up to three years, during which multiple therapists work with patients. Patient recovery is influenced by many components beyond therapy sessions, including adherence to home exercises, the use of the affected limb by patients in everyday activities, and the support available for patients during daily living. Because of this variability, the physical therapy field has developed structured and formalized assessment tools that are administered at regular intervals during the lengthy process of therapy to track patient progress and inform therapy customization. The overarching goal of therapy is to support the functional recovery of patients and facilitate reintegration into daily life activities. Thus, therapy assessment tools are expertly structured to assess progress toward functional recovery. The Action Research Arm Test (ARAT) (Yozbatiran et al., 2008) is a clinically validated tool for assessing the upper extremity of stroke survivors. It comprises 19 individual exercises that are generalizable to the functional use of the upper limb during daily living. Therefore, improvements in the ARAT are expected to generalize to improved overall functionality.

Our AI development team, in close collaboration with eight therapists at a major rehabilitation hospital, identified the following common goals for developing human-AI collaboration processes to support augmented ARAT assessment. Augmented assessment would free therapists to focus on delivering therapy. It could also increase the standardization and granularity of observations during therapy without requiring extra time or effort from therapists. Increased standardization and granularity through automation that is intuitively accessible to therapists would make assessments portable across therapists and clinics and inform effective therapy customization. In this context, human-AI collaboration aims to empower and assist (augment) the expert in performing the complex task of physical therapy. It does not aim to replace the expert.

Our second co-design step was to define a nested network representation of the ARAT that would be computable as a DBN. We first co-defined the observable elements of the network. The ARAT has a highly codified environment and tools. It includes a table and a chair, with a shelf of specific dimensions on the table, and a mat of specific dimensions placed in a specific location on the table. The mat includes drawings for placing the ARAT objects and the hands of the patients in standardized positions. The ARAT uses standardized objects with different affordances for each of the 19 exercises. The instructions for each exercise are defined in a manual as a precise interaction between the patient's body and the standardized environment (McDonnell, 2008). Each exercise has a well-specified goal, which is articulated to the patient before they begin the exercise. For example, please start with your arm on the edge of the table, move to grasp the “specific object” at the “specific location” with “specific fingers,” and move it to a “specific location” within 1 min (the “specifics” change per exercise). Thus, defined and observable E-A interactions can be linked to the specified Goals (G) of each exercise.

The ARAT manual (McDonnell, 2008) includes specific instructions on what the therapist needs to observe for the standardized assessment of each of the 19 activities. Based on these observations, the therapist uses a well-defined scoring rubric (included in the manual) to assign a score of 0–3 to each exercise. The rating of each exercise, along with the rating rubric, provides explicit and observable features of Meaning extraction (M) by the therapist that can be connected to specific E, A, and G elements for each exercise assessment. The individual scores across the 19 exercises are summed to yield a total score of 0–57 for the entire complex task (the ARAT), thereby providing an explicit and observable EAGM network for the entire task. Repeated ARAT sessions during long-term therapy allow tracking of the network's evolution across sessions. The ARAT structure can thus be readily aligned with the four information aggregation structures of the DBN: (i) the instance: the smallest unit of high quality observation of the E and A elements of the ARAT, (ii) an individual action of the complex task with a well-defined start and endpoint: an individual exercise of the ARAT, (iii) one performance of the complete complex task—a complete ARAT assessment, and (iv) a sequence of sessions of the complex task—the multiple ARAT sessions.

However, most elements of ARAT performance are hidden. The therapist brings hidden embodied knowledge from training, practice, and everyday living to the assessment process. The detailed meaning of how the ARAT assessment informs the adaptation of therapy to address individual patient needs is embodied in the therapist's expert practice and interactions with each patient and is also hidden (Winstein et al., 2016; Krakauer, 2006). The patient's needs for functional recovery in daily life and their motivations for recovery may be partially revealed to the therapist through their embodied interactions with patients, but in most cases, they remain largely hidden. Furthermore, therapists, like all human beings active in the wild (Brown et al., 2011), observe detailed statistical features of the E and A interactions that are not expressible as a linear sequence of specific individual elements. After all, a significant part of human sensorimotor daily experience (proprioception, motor planning, auditory, and visual scene analysis) is actually statistical and activated through embodied perception action cycles and thus hidden (Wolpert, 2007; Ahlheim et al., 2014).

### Instantiating the DBN model for automated ARAT assessment

**Figure 3:**
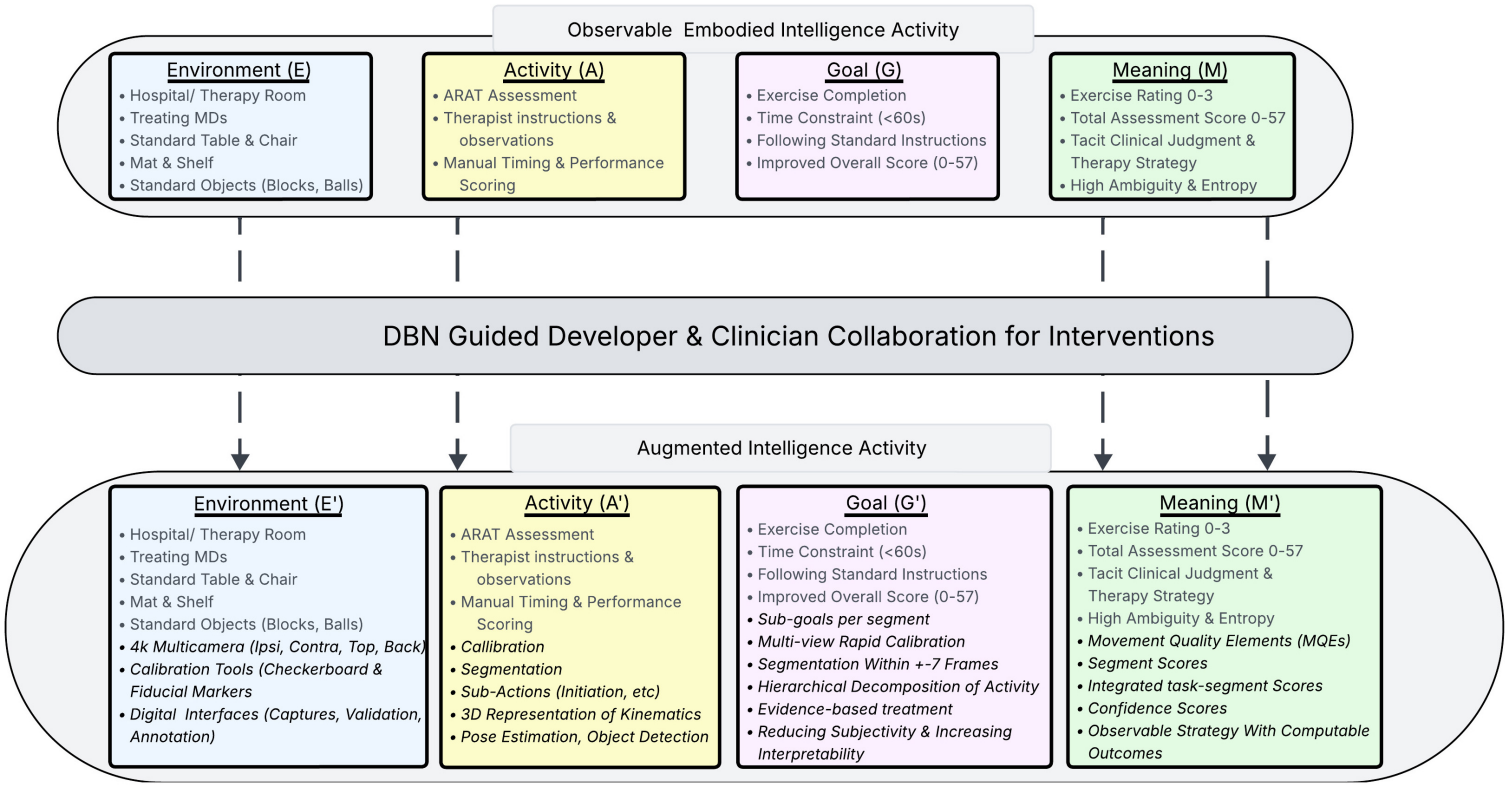
Transformation of the ARAT assessment into an Augmented Intelligence Activity. The framework illustrates the translation of the standard clinical observation loop [**(Top)** Observable embodied intelligence activity with Environment E, Activity A, Goal G, Meaning M] through a DBN-guided co-design process [**(Middle)** DBN Guided Developer & Clinician Collaboration for Interventions] into a digitized, computational environment [**(Bottom)** Augmented intelligence activity with enriched *E*′, *A*′, *G*′, *M*′ layers]. Each enhancement passes through the DBN's gated pipeline—compatibility, coherence, and observability checks—before validation by clinicians and developers leads to permanent network updates.

We now ground the generic, bidirectional DBN within our Augmented ARAT Assessment system. Figure 3 provides a comprehensive visualization of this transformation, illustrating the journey from standard clinical observation (top panel) through DBN-guided co-design (middle panel) to an augmented intelligence approach (bottom panel). Critically, this transformation is not a single redesign but an *iterative process* in which each proposed computational enhancement must pass through the DBN's gated pipeline—compatibility check, coherence gate, and observability gate—before clinicians and developers validate its integration into the permanent network structure.

In the initial version of DBN, Environment symbols *S*^*E*^ encode the standardized ARAT setup, Activity symbols *S*^*A*^ encode patient activity, Goal symbols *S*^*G*^ encode the immediate goal of each ARAT exercise, and Meaning symbols *S*^*M*^ encode the explicit ratings provided by the therapist. As the co-design of the Augmented ARAT Assessment system proceeds, the DBN is gradually enriched through human and computational insights. In the following sections of the paper, we outlined each of the core additions that transformed human assessment of the ARAT into an augmented (human-AI collaborative) assessment. We identified the problem the addition aims to address, specified the new codes (lexicon updates) produced by the addition, described the DBN factors the addition modifies, outlined the measured/expected effects on *coherence and visibility gain*, and presented the resulting DBN posterior updates that lead to uncertainty reduction in the assessment of the ARAT. In general, we expect to see the following changes in entropy (H) and coherence ($C_{\text{agg}}$) for an added component *x*:


$$\begin{array}{r} \Delta H^{X}\equiv H_{\text{before}}\left(X\right)\;-\;H_{\text{after}}\left(X\right)\;\left(\geq 0\right)\;\text{and }\Delta C_{\text{agg}}\equiv \\ \underset{X\in \left\{\text{act},\text{task},\text{seq}\right\}}{\sum }w_{X}(C_{X}^{\text{after}}-C_{X}^{\text{before}})\left(\geq 0\right), \end{array}$$

#### Multi-view capture with expert controls: enriching environment (*E*→*E*′)

Digitization and symbolization of the Environment and Activity elements of the ARAT require capture methods that do not interfere with the patient or therapist activities. Therapists move during each exercise to improve their detailed observation of its various elements. Therapists do not denote the connections between their movements and the Activity and Environment elements they observe. The features and connections among the therapist, patient, and Environment (E) interactions need to be computed from a multi-view capture of the ARAT. As these connections were not observable, the DBN initially could not establish strong *c*4 edges. The single-view system left many patient movements ambiguous, flagging high residuals (many near-miss κ_*t*_ patterns) and low confidence in symbolization. This prompted the developers to propose multi-view capture, along with rapid calibration protocols, as a candidate to enhance E–A layer capture.

The multi-view system proposes new Environment symbols V={ipsi,contra,transverse,posterior} that are compatibly connected to existing *A* patterns (certain views are systematically better for certain activities) and existing *G* patterns (specific goals require specific viewing angles). The custom views will help produce more detailed and more confident assessments (M). The compatibility check passed because the proposal enriched the network without contradicting established *E*–*A*–*G*–*M* patterns. The search/attention policy assigns control *a*_*t*_ = (*v*_*t*_, *z*_*t*_, *s*_*t*_) for view, zoom, and half-speed playback. The integration of the views, kinematic and visual features extracted using computational models, and different viewing features yielded the following effects in our proposed bi-directional DBN.

(i) *Observation model* *p*(*Obs*_*t*_∣*a*_*t*_, *e*_*t*_): Multi-view improves two aspects of the likelihood: pixels (fewer occlusions, better SNR) and 3D kinematics (better geometry for triangulation). Since 3D kinematics are computed from video, we model them as conditionally dependent on pixels given (*a*_*t*_, *e*_*t*_). The full observation factor separates pixel-based evidence from kinematic reconstruction:


$$\begin{array}{l} \;p({\text{Obs}}_{t}|a_{t},e_{t})=p({\text{Video}}_{t}|v_{t},z_{t},s_{t},e_{t})\cdot p({\hat{X}}_{t},\Sigma_{t}^{\text{kin}}|\\ {\text{Video}}_{1:t},v_{1:t},e_{t}), \end{array}$$ (26)

with ${\hat{X}}_{t}$ the reconstructed pose/hand–object state. As the kinematic covariance $\Sigma_{t}^{\text{kin}}$ shrinks, downstream inference becomes sharper.

(ii) *Symbolizers* $p\left(S_{t}^{A}|{\text{Obs}}_{t}^{A}\right)$, $p\left(S_{t}^{M}|{\text{Obs}}_{t}^{M}\right)$: When multi-view reduces $\Sigma_{t}^{\text{kin}}$, features become less noisy, and the posteriors $p\left(S_{t}^{A}\right)$, $p\left(S_{t}^{M}\right)$ become less entropic (more confident). This directly reduces uncertainty at the symbol level and propagates it through the DBN.

(iii) *c*4 *builder* $p\left(r\in R_{t}^{c4}\right)$: The cross-layer edge *r* = (*s*^*E*^, *s*^*A*^, *s*^*G*^, *s*^*M*^) is activated by a sigmoid that combines local compatibility with historical coherence.


$$\begin{array}{l} \kappa_{t}^{\prime }\left(r\right)=\\ \text{sigmoid}(\underset{\text{base}}{\underset{︸}{\phi_{0}\left(s^{A},s^{G},s^{M}\right)}}+\underset{\text{preferred view for action}}{\underset{︸}{\phi_{\text{view}}\left(s^{E},s^{A}\right)}}+\underset{\text{evidence quality}}{\underset{︸}{\phi_{\text{kin}}(\Sigma_{t}^{\text{kin}})}}), \end{array}$$ (27)

$\phi_{\text{view}}\left(s^{E},s^{A}\right)$ learns that some views are systematically better for certain actions (e.g., contralateral for pre-shaping and ipsilateral for termination). $\phi_{\text{kin}}\left(\Sigma_{t}^{\text{kin}}\right)$ rewards low uncertainty, e.g., $\phi_{\text{kin}}=-\alpha \log\det\Sigma_{t}^{\text{kin}}$. The coherence prior $\psi_{t}^{\text{coh}}$ comes from aggregated edge histograms and up-weights historically consistent quadruples. Together, multi-view raises both κ_*t*_(*r*) (better match & evidence) and $\psi_{t}^{\text{coh}}\left(r\right)$, as view–activity pairs (e.g., contralateral view for pre-shaping and ipsilateral for termination) show stable co-occurrence patterns across actions, tasks, and sequences. The coherence prior $\psi_{t}^{\text{coh}}$ increased for historically consistent view–action patterns, yielding $\Delta C_{\text{agg}}>0$ and sharper DBN posteriors. Multi-view capture enabled the discovery of new quadruples that were previously unobservable. With improved geometry for 3D triangulation and fewer occlusions, the system could now build *c*4 edges connecting specific views to specific activities, goals, and meanings: ${nGain}_{c4}^{E}>0$.

A lightweight *policy*, such as the recommended view *v*_*t*_ by action (from π^*coh*^), exposes *z*_*t*_ and *s*_*t*_ = 0.5 × that are introduced when hand–object events are predicted. The policy objective given by Equation 4 now includes zoom/speed. There is no change in the lexicon, and the improvement comes from better-conditioned evidence for existing symbolizers. The improvement will generate faster disambiguation with fewer redundant looks, satisfying the conditions: $\Delta H^{{\left(g,m\right)}_{t}}>0$ at the same or lower action cost; $\Delta C_{\text{agg}}>0$ via more consistent, high-quality edges across repeated uses. After the multi-view system demonstrated stable performance across multiple assessment sessions (Δ*t*), clinicians and developers reviewed the accumulated evidence. Clinicians confirmed that the automated view selection aligned with their tacit viewing strategies and that greater control over payback speed would improve video capture for review and, therefore, assessment. The developers verified that the 3D kinematic reconstruction improved the symbolizer's confidence. Upon validation, the Environment layer was permanently updated:


$$\Sigma_{\text{after}}^{E}=\Sigma_{\text{before}}^{E}\cup V,\;E\to E^{\prime }$$

#### Action segmentation: enriching activity (*A*→*A*′)

Multi-view capture and analysis by AI designers and therapists have begun to reveal a critical yet tacit and non-standardized approach to activity segmentation. Therapists use a generalizable segment vocabulary that enables meaningful assessment of specific movement components across different ARAT exercises and can inform therapy customization (e.g., targeting the reduction of trunk compensation during movement initiation across all types of upper-limb activity). Using computational methods and expert input to reveal and standardize segmentation could add explicit, observable EAGM connectivity to the network and improve posteriors. The *c*4 builder showed stable co-occurrence clusters of partial tuples [e.g., (*s*^*E*^, *s*^*A*^, *, *)] at specific temporal phases, but these patterns did not connect to explicit Goal or Meaning symbols. This triggered the proposal mechanism to surface explicit segmentation as a candidate enhancement.

It would provide an additional time-aggregation bracket (*sub-action*) that bridges *instant* and *action* aggregate assessments across all ARAT exercises and support tuning EAGM assessments based on the type of sub-action (segment) being performed. The clinicians defined an initial segmentation vocabulary, which was gradually refined through co-design activities that leveraged computational insights from the DBN. The final vocabulary comprises four segments mapped to subactions in the DBN: *initiation and progression, termination, manipulation and transportation*, and *place and release*. This vocabulary was proposed as a candidate enhancement to the Activity layer.

The developers added the *sub-action* vocabulary within *A* and an automated segmenter $Seg\left(X_{1:T}^{A};\theta_{seg}\right)$ that produces spans $\left(t_{j}^{s},t_{j}^{e}\right)$ with types $s_{j}^{A}\in \Sigma^{A}$ and confidence $\pi_{j}^{A}$. Initially, the activity layer used the per-frame activity classifier, $p\left(S_{t}^{A}=s|X_{t}^{A}\right)$ with a likelihood $\prod_{t}p\left(S_{t}^{A}|X_{t}^{A}\right)$. Our segmentation component replaces this with a structured factor $p\left(S_{1:T}^{A}|X_{1:T}^{A},\Sigma^{A};\theta_{seg}\right)$ that enforces span consistency from the automated segmenter:


$$\begin{array}{r} p(S_{1:T}^{A}|X_{1:T}^{A},\Sigma^{A};\theta_{seg}(\propto \underset{\text{frame evidence}}{\underset{︸}{\overset{T}{\underset{t=1}{\prod }}\theta_{t}^{A}[S_{t}^{A}]}}\;\times \\ \underset{\text{temporal/ordering prior}}{\underset{︸}{\overset{T}{\underset{t=2}{\prod }}\text{Ψ}(S_{t-1}^{A},S_{t}^{A})}}\;\times \;\underset{\text{span consistency from the segmenter}}{\underset{︸}{\overset{J}{\underset{j=1}{\prod }}(\pi_{j}^{A}1\left\{S_{t_{j}^{s}:t_{j}^{e}}^{A}\equiv s_{j}^{A}\right\}+\left(1-\pi_{j}^{A}\right))}}. \end{array}$$ (28)

The *frame evidence* maintains the information from the per-frame classifier (what this instant looks like). The *temporal/ordering prior* $\text{Ψ}\left(S_{t-1}^{A},S_{t}^{A}\right)$ encodes legal progressions and smoothness (e.g., Initiation→Progression→Termination), discouraging implausible jumps. A duration bias can be included by augmenting Ψ (semi-Markov) to prefer sub-actions that last multiple frames. *Span consistency* utilizes the automated segmenter's proposal $B=\left\{\left(t_{j}^{s},t_{j}^{e},s_{j}^{A},\pi_{j}^{A}\right)\right\}$ as a soft constraint: within the proposed span $\left[t_{j}^{s},t_{j}^{e}\right]$, the labels are encouraged to equal $s_{j}^{A}$, with strength controlled by confidence $\pi_{j}^{A}\in \left[0,1\right]$. If $\pi_{j}^{A}$ is high, the span behaves like a nearly fixed sub-action of type $s_{j}^{A}$; if it is low, the model falls back to frame evidence and Ψ.

Within a span $\left[t_{j}^{s},t_{j}^{e}\right]$, the posterior over $S_{t}^{A}$ collapses onto the proposed type $s_{j}^{A}$ (when $\pi_{j}^{A}$ is high), so the entropy $H\left(S_{t}^{A}\right)$ drops across the span. Stable $S_{t}^{A}$ increases the *c*4 compatibility $\kappa_{t}\left(s^{E},s^{A},s^{G},s^{M}\right)$ for the appropriate view–sub–action–goal–meaning, where each segment type is connected to specific Environment configurations (camera views optimal for that segment), specific Goal interpretations (what needs to be achieved during that segment), and specific Meaning indicators (what quality elements matter during that segment). Because the same quadruples activate reliably across repetitions of similar sub-actions in the 19 exercises of the complex task, the aggregate histograms that form π^*coh*^ concentrate on clinically consistent patterns, which increases the coherence potential $\psi_{t}^{\text{coh}}$ used by the builder. The *c*4 builder achieves many tighter action–goal–view alignments.

Here is one example of the new four-layer connections. If the segmenter proposes frames 30:60 as hand pre-shaping with confidence π^*A*^ = 0.9, the structured factor will assign $S_{30:60}^{A}\approx \text{pre-shaping}$ (as a child of the Progression sub-Action) unless the frame evidence is overwhelmingly contradictory. A more confident sub-Action recognition will leverage the multi-view *c*4s to establish a contralateral, zoomed-in view as the preferred view for the frames. This will decrease occlusions, enhance sampling (number of pixels) per tracked finger, and reduce frame jitter. It enables a new sub-goal (assessment of hand-shaping pre-grasp) that can be reliably assessed across all exercises (new Meaning). It thus enables new *c*4 quadruples connecting segments to views, goals, and meanings that were previously unobservable: ${nGain}_{c4}^{A}>0$. This allows the *c*4 builder to consistently activate the correct quadruples—producing sharper posteriors for the orientation (*g*_*t*_, *m*_*t*_) and more confident downstream scoring.

After demonstrating stable segmentation across multiple sessions, clinicians confirmed that the four-segment vocabulary captured their tacit assessment structure. Developers verified that automated boundary detection achieved ±7 frame accuracy. The Activity layer was permanently updated:


$$\begin{array}{l} \Sigma_{\text{after}}^{A}=\Sigma_{\text{before}}^{A}\\ \cup \left\{\text{initiation},\text{termination},\text{manipulation},\text{release}\right\},\;A\to A^{\prime } \end{array}$$

#### Segment-focused movement quality: enriching activity and expanding goals (*A*→*A*′, *G*→*G*′)

With the inclusion of the segment (sub-action) vocabulary, the DBN revealed another gap; the Meaning layer remained ambiguous because different movement quality elements (MQEs) have varying relevance depending on the segment being performed. The system showed high entropy in $S_{t}^{M}$ despite improved Activity symbolization, prompting a proposal for segment-focused MQE weighting. Based on prior collaborative work (Kelliher et al., 2020), we asked the experts to define a small number of movement quality elements that they focus on per type of segment during their practice. Once the MQEs were developed, the team concluded that they introduced a new level of granularity to the observation and assessment of ARAT. The therapists performed such assessments intuitively but could conduct them accurately, explicitly, and in a standardized manner only with the aid of computation. This is a clear example of how EAGM can transform into E'A'G'M', as shown in Figure 3, through the addition of new elements that significantly enhance the explicit cross-layer connectivity. The translation between human observation and AI-enhanced observation is performed by the DBN.

Based on the movement quality elements (MQE) for sub-action vocabulary, symbolizers σ_*A*_, σ_*M*_ were updated to classify into the refined lexicons:


$$S_{t}^{A}\sim p_{\theta }(\cdot |{\text{Obs}}_{t}^{A},\;\Sigma^{A}),\;S_{t}^{M}\sim p_{\phi }(\cdot |{\text{Obs}}_{t}^{M},\;\Sigma^{M}).$$

We also introduced a *focus prior* $\pi_{\text{focus}}(s^{M}|s^{A}($ that defines which MQEs *m* receive attention given segment *s*^*A*^, which up-weights the expert-nominated MQEs for the current sub-action and augments the updated compatibility from Equation 27 to recognize these focused co-occurrences, while the coherence potential $\psi_{t}^{\text{coh}}$ accumulates support for the resulting *c*4 edges across actions, tasks, and sequences. For example, when a patient struggles with trunk compensation during initiation, this consistently predicts lower exercise scores. The segment-specific MQE weights accumulated stable evidence: $\Delta C_{\text{agg}}>0$


$$\begin{array}{l} \kappa_{t}^{\prime \prime }\left(s^{E},s^{A},s^{G},s^{M}\right)=\\ \text{ sigmoid}(\phi_{0}\left(s^{A},s^{G},s^{M}\right)+\phi_{\text{view}}\left(s^{E},s^{A}\right)+\phi_{\text{kin}}(\Sigma_{t}^{\text{kin}})\\ \;+\lambda_{\text{focus}}\log\pi_{\text{focus}}\left(s^{M}|s^{A}\right)). \end{array}$$ (29)

This targeted weighting aligns the machine's meaning space with clinicians' mental models and measurably reduces ambiguity with sharper MQE posteriors ($\Delta H\left(S_{t}^{M}\right)<0$) and clearer orientation (Δ*H*((*g, m*)_*t*_) < 0) from the DBN. Thus, it increases cross-layer visibility and stability across action/task sequences.

#### Expert-defined sub-goals and related meaning: enriching goal and meaning (*G*→*G*′, *M*→*M*′)

The introduction and refinement of a sub-action vocabulary that is valid across all individual exercises of the complex task (ARAT), along with the attachment of a few highly weighted movement quality elements to each sub-action, increased the explicit and observable elements of the DBN. However, this increased observability did not produce the reduction in assessment entropy predicted by the DBN. Our discussions had already identified potential causes of this problem. The clinicians noted that they began to predict the overall meaning (and associated ratings) of an individual ARAT exercise from the performance of its constituent segments. For example, the “Initiation and Progression” segment needs to be completed appropriately for the hand to be able to start the “Terminate” segment for a specific object of an ARAT exercise. We thus concluded that segments (sub-actions) should be assigned specific sub-goals and associated meanings (ratings) that can be aggregated consistently into the overall Goal and Meaning of each ARAT exercise (action).

The clinicians proposed that since each exercise was rated between 0 and 3 using a standardized rubric, the segments should also be rated on the same scale using a similar rubric. Given the speed of each exercise and the many elements to be assessed, the clinicians suggested that the movement quality elements be assessed using a binary classification: 1 for appropriate and 0 for not appropriate. The term “appropriate” connects the Meaning of the assessment of specific movement quality elements to the Meaning of the assessment of a specific sub-goal; in other words, the performance of a movement quality element is appropriate for the segment during which it is performed. In turn, the consistent ratings of segments and exercises connect the Meaning of sub-goal assessment to the Meaning of Goal assessments. It allows the system to derive Augmented Meaning (*M*′) not as an ambiguous overall rating but as a composite of explicit, confidence-weighted scores of Exercises, segments, and Movement Quality Elements (MQEs).

Building on the focus-weighted compatibility from the previous subsection (Equation 29/$\kappa_{t}^{\prime \prime }$), we now incorporate expert annotations that make two aspects explicit at the sub-action level *j*: (i) an *ordinal sub-goal rating* Ỹ_*j*_ ∈ {0, 1, 2, 3}; (ii) a *binary status* *m*_*j, q*_ ∈ {0, 1} for each focused movement-quality element (MQE) $q\in F\left(s_{j}^{A}\right)$ tied to the sub-action type $s_{j}^{A}$. These serve as observational factors and drive both symbol learning and *c*4 edge formation.

Let *h*_*j*_ be a learned embedding (from multi-view pixels/kinematics) pooled over instants $t\in I_{j}$ of sub-action *j*:


$$\begin{array}{l} h_{j}=f_{\text{tr}}({\left\{X_{t}^{\text{pix}},X_{t}^{\text{kin}}\right\}}_{t\in I_{j}})\in {\mathbb{R}}^{d}, \end{array}$$ (30)

and $H_{T}=Pool\left({\left\{h_{j}\right\}}_{j\in U_{T}}\right)$ be the action-level embedding. During training, expert labels supervise Equations 31–33, but at inference, these factors provide calibrated evidence to the DBN that is predicted from automated algorithms:


$$\begin{array}{l} p\left({\tilde{Y}}_{j}|h_{j}\right)=\sigma \left(\gamma_{k}-u^{⊤}h_{j}\right);\;k=0,1,2, \end{array}$$ (31)

$$\begin{array}{l} p\left(Y_{T}|H_{T}\right)=\sigma \left(\delta_{k}-v^{⊤}H_{T}\right);\;k=0,1,2, \end{array}$$ (32)

$$\begin{array}{l} p\left(m_{j,q}=1|h_{j}\right)=\sigma (w_{q}^{⊤}h_{j}+b_{q}),\;q\in F\left(s_{j}^{A}\right). \end{array}$$ (33)

At each instant $t\in I_{j}$, we form *per-MQE* quadruples $r_{t,j,q}=\left(s_{t}^{E},\;s_{t}^{A},\;G_{j},\;m_{j,q}\right)$, where *G*_*j*_ denotes the (latent/predicted) sub-goal code consistent with Ỹ_*j*_. We update the focus-aware compatibility from Equation 29


$$\begin{array}{l} \kappa_{t}^{\prime \prime \prime }\left(s_{t}^{E},s_{t}^{A},G_{j},m_{j,q}\right)=\\ \text{ sigmoid}(\phi_{0}\left(s_{t}^{A},G_{j},m_{j,q}\right)+\phi_{\text{view}}\left(s_{t}^{E},s_{t}^{A}\right)\\ \;+\phi_{\text{kin}}(\Sigma_{t}^{\text{kin}})+\lambda_{\text{focus}}\log\pi_{\text{focus}}\left(q|s_{t}^{A}\right)), \end{array}$$ (34)

and build edges with coherence-weighted activation using the updated $\psi_{t}^{\text{coh}}\left(r_{t,j,q}\right)=\log\frac{\pi_{t}^{\text{coh}}\left(r_{t,j,q}\right)}{1-\pi_{t}^{\text{coh}}\left(r_{t,j,q}\right)}$.

With this update to the proposed DBN, each expert label (Ỹ_*j*_, {*m*_*j, q*_}) becomes an explicit code and adds valid, focus-compatible candidates for *c*4 edges (more observable cross-layer structure; ${nGain}_{c4}^{G}>0$, ${nGain}_{c4}^{M}>0$). Furthermore, the standardized sub-goal rubrics and binary MQEs shrink hypothesis spaces, tightening posteriors for $S_{t}^{G}$, $S_{t}^{M}$, and (*g, m*)_*t*_ ($\Delta H\left(S_{t}^{G}\right)<0$, $\Delta H\left(S_{t}^{M}\right)<0$, Δ*H*((*g, m*)_*t*_) < 0). Thus, action/task/sequence histograms over {preferred view, sub-action, Ỹ_*j*_, *m*_*j, q*_} concentrate on reusable motifs, increasing ψ^*coh*^ for those edges and making the top-down prior more predictive regarding subsequent cases. With the clinicians validating that the hierarchical goal-meaning structure captured their reasoning process, both the Goal and Meaning layers were permanently updated:


$$\begin{array}{r} G\to G^{\prime }\text{with sub-goals per segment},\\ M\to M^{\prime }\text{with segment ratings and binary MQEs} \end{array}$$

#### Computational ensemble development for automated scoring and quality inference

With all EAGM layers enriched through the gated validation process, we developed an AI ensemble that operationalizes the complete transformation. This ensemble connects the DBN to pose estimators, 3D kinematics extractors, and joint transformer/HBM predictors (Ahmed et al., 2024), producing calibrated predictions that feed back into the DBN's gated pipeline.

First, multi-view 4K videos are processed through 2D pose and hand keypoint estimators (Ahmed et al., 2021) and hand-object contact detectors (Lee et al., 2025) to extract body and hand joint coordinates and object locations. View-synchronized triangulation produces 3D kinematics $\left(X_{t}^{\text{kin}}\right)$ that populate the observation channels: ${\text{Obs}}_{t}^{E}$ (active view features), ${\text{Obs}}_{t}^{A}$ (motion and velocity cues), and ${\text{Obs}}_{t}^{M}$ (hand aperture, wrist orientation, trunk compensation, smoothness, and accuracy in relation to the score).

Second, we trained predictors on clinician-validated data: (i) a multi-view transformer for sub-action span detection and (ii) a fusion of transformer-based activity recognition with hierarchical Bayesian models (HBM) (Ahmed et al., 2024) trained on multi-rater sub-action/action scores with MQEs.

The ensemble outputs replace or augment the learned symbolizers:


$$\begin{array}{r} p\left({\tilde{Y}}_{j}|h_{j}\right)\;\leftarrow \;\bar{P}\left({\tilde{Y}}_{j}\right),\;p\left(m_{j,q}|h_{j}\right)\;\leftarrow \;\bar{p}\left(m_{j,q}=1\right),\\ p\left(Y_{T}|H_{T}\right)\;\leftarrow \;\bar{P}\left(Y_{T}\right). \end{array}$$ (35)

where $\bar{P}$ and $\bar{p}$ are fused predictions from the transformer and HBM in the logit space. Crucially, the ensemble's predictions are not directly presented to clinicians. Instead, they enter the DBN as candidate *c*4 edges that must pass through the same gated pipeline: compatibility with existing patterns, coherence over time, and positive observability gain. Predictions that fail these gates are flagged for refinement or added to the error list.

The final compatibility equation for the ARAT assessment, updated through the complete co-design process, is:


$$\begin{array}{l} \kappa_{t}^{\prime \prime \prime \prime }\left(s_{t}^{E},s_{t}^{A},G_{j},{\hat{m}}_{t,j,q}\right)=\\ \text{ sigmoid}(\underset{\text{base}}{\underset{︸}{\phi_{0}\left(s_{t}^{A},G_{j}\right)}}+\underset{\text{preferred view}}{\underset{︸}{\phi_{\text{view}}\left(s_{t}^{E},s_{t}^{A}\right)}}+\underset{\text{kinematic quality}}{\underset{︸}{\phi_{\text{kin}}(\Sigma_{t}^{\text{kin}})}}\\ \;+\underset{\text{MQE focus}}{\underset{︸}{\lambda_{\text{focus}}\log\pi_{\text{focus}}\left(q|s_{t}^{A}\right)}}+\underset{\text{MQE evidence}}{\underset{︸}{\phi_{mqe}\left({\hat{m}}_{t,j,q}\right)}}), \end{array}$$ (36)

where each term reflects an enhancement that has passed through the gated pipeline and has been validated by clinicians and developers, the result is a transformed EAGM network, where Environment (*E*′) includes multi-view capture infrastructure with automated view selection, Activity (*A*′) includes explicit segmentation with a temporal structure, Goal (*G*′) includes hierarchical sub-goals with segment-level objectives, and Meaning (*M*′) includes segment ratings, binary MQEs, and confidence scores. The “High Ambiguity/Entropy” that originally characterized the Meaning layer has been replaced by “Observable strategy with computable outcomes”—not by imposing a rigid structure on clinical judgment but by making tacit knowledge explicit through a principled, evidence-driven process that preserves alignment with expert practice.

### Study set up and outcomes

Our automated ARATs assessment tool was deployed at a major rehabilitation hospital in the Midwest, where five clinicians used our four-camera multi-view capture system to record routine ARATs assessments of 105 stroke survivors over six months. The clinical study was approved by the IRB, and all participants (patients and clinicians) provided informed consent before data collection, workshops, and group discussions for the co-development of the system. The study produced a dataset of 1,800 exercise performances. The automated segmentation algorithm reached 91% agreement with human boundaries within ±7 frames. The exercise and segment clips were then rated by five expert clinicians using the standardized assessment vocabulary co-developed. Inter-rater agreement among the clinicians was substantial but also exhibited uncertainty characteristic of decisions in complex tasks, which became more pronounced as the granularity increased. Kappa scores for inter-rater agreement for exercises and segments were (κ = 0.748) and (κ = 0.699) respectively, but (κ < 0.600) for movement quality features (MQEs).

We then trained the ensemble models to automate ARAT exercise assessment. The automated assessments matched clinician ratings at 90.8% at the exercise level, 93.1% at the segment level, and 90.6% at the movement quality level for cases where clinicians' ratings were in agreement. With the addition of statistical probabilistic modeling from the HBM and calibrated posteriors aligned with sub-action contexts, the ensemble models disambiguated 92% of cases in which clinicians' ratings disagreed. We validated 400 machine-generated assessments with four expert clinicians who were not part of the system's co-design process. The clinicians agreed with the automated ratings at 90.8%, 93.1%, and 90.56% on the exercise, segment, and movement quality levels, respectively. They also reported a strong fit with their clinical practice. They indicated that the customization of therapy by clinicians can be well informed by access to automated assessments that link an exercise rating to details of the exercise's performance and can illustrate these connections through synchronized multi-view and time-variable displays playback. Details of the automated assessment models and validation study results will be published in an upcoming paper. We also plan to release de–identified data from the study after that publication.

## Discussion

The ARAT project illustrates our co-design approach to human-AI collaboration for the emergence of augmented intelligence in complex task performance. Engineers and therapists establish common goals for the project and then begin to collaborate on the components. The therapists provide the explicit symbols for the first version of the EAGM network for Augmented Therapy Assessment. Engineers set up the infrastructure to digitize the explicit elements. This allows for the first computation of the EAGM as a DBN. The DBN identifies opportunities for newly added digital elements or therapist-provided information to enhance the network's observability and confidence. Suggestions can be quickly tested and refined as the DBN makes connections or identifies missed connections between new elements and the nested network, which is observable and quantifiable. The increased observability of the network makes experts' tacit knowledge explicit and guides computational enhancements that improve both explicit and tacit practices among clinicians. The DBN provides the computational agents with concrete, in-the-loop proposals (“switch to contralateral camera, zoom on digits, extract finger relations to object”) that mirror expert habits. The appropriate AI agent(s) can then operationalize the choice and deliver the specific measurements requested by the DBN. In turn, the DBN can justify a computational assessment recommendation to the human expert with a short, symbolic rationale: “the contralateral view of hand pre-shape for secure grasp co-occurs with inadequate aperture; the probability is high given past sessions.”

This AI reasoning and its outcomes are preferable to opaque saliency maps; it speaks the expert's language and is auditable. Experts can confirm and/or correct the computational ensemble predictions and recommendations with minimal effort, thereby integrating them into routine workflows. If the AI recommendation is accepted, the integration of the recommendation into future therapy strategies is left to the expert. When a patient returns for their next ARAT assessment, the computational ensemble can compute detailed changes from the previous sessions and help further inform the therapist about the effectiveness and/or direction of adaptation. The collaborative architecture allows the human performer(s) to readily receive low information, high-pertinence input from the AI agents and amplify the effect of this information to significantly enhance performance. In practice, this means a computational ensemble that keeps up with the expert, offering well–timed, well–founded suggestions that reduce ambiguity and enhance performance on complex tasks without increasing sensorimotor or cognitive load. As the human-AI collaboration advances, the human expert's behavior changes, and the computational ensemble keeps up with those changes.

It may seem counterintuitive that as the amount of information processed by human-AI collaboration increases and becomes more complex, the entropy in performance assessment decreases while confidence in future planning increases. We propose that this occurs because the computational agents are not attempting to increase the degree of explicit standardization in task performance. We have already shown in earlier sections that complex task performance relies on a combination of standardized explicit procedures and tacit embodied procedures that are sensitive to the ever-changing complexities of the task. Therefore, the probabilistic nature of DBNs enables the translation of computational contributions into recommendations for evidence-based adaptation of performance by the embodied intelligence of the expert.

The application of computational ensembles to the ARAT assessment can be adapted to other complex task contexts. For example, in the ARAT, we enhanced observation through multi–view capture, which was linked to the development of a custom sub–action vocabulary. In surgery, one might add laparoscopic view modes linked to procedural phases. In sports, one may add broadcast angles and micro–technique phases. The abstractions of the DBN (explicit symbols, *c*4 edges, bidirectional inference, coherence priors, discovery/gating) remain constant, and the ensemble of AI modules is domain–specific. In each case, the DBN is the invariant translator; the AI ensemble is the adaptable measurement layer; the co–design loop ensures both remain aligned with expert intent for emerging human-AI collaboration.

## Limitations and future work

Our approach emphasizes scalability and generalizability; yet both remain open challenges that we address through several research directions. First, the co-design program we advocate—iterative, participatory, and evidence-driven—requires sustained engagement with domain experts; this yields durable artifacts but does not scale immediately across many sites or tasks. Second, while the bidirectional DBN is domain-agnostic, each deployment requires porting the explicit, observable lexicons (Environment, Activity, Goals, Meaning) of the target complex task, as well as their symbolizers. This means that the methodology is best suited to complex tasks that have been at least partially formalized through expert practice. The methodology relies on these expert practitioners to map AI insights into enhanced practice. For example, in our physical therapy application, it is up to expert clinicians to map augmented assessment into customized therapy. Furthermore, we have tested our methodology only in asynchronous practice; applications where AI agents act synchronously with experts will require significant enhancements to real-time inference and interaction design.

A key opportunity for scalability lies in the transferability of evolved DBN networks. Once a network has been symbolized and validated for a particular complex task, it can serve as a *pretrained model* for related tasks—similar to how pretrained neural networks provide useful initializations for similar domains. This evolved network could initialize DBNs for related functional assessments in which the underlying EAGM structure exhibits significant overlap. In the context of rehabilitation, this would apply to other assessment instruments (e.g., the Wolf Motor Function Test and the Box and Blocks Test), to the assessment of therapy sessions, and to the development of training assistants for less-experienced therapists. This approach could substantially reduce the burden of co-design in new deployments while preserving the principled, evidence-driven nature of network evolution.

As a next step toward scalability, we will work with our partner clinic to build Explainable Augmented Automated Rehabilitation Assistant (xAARA) to assess therapy sessions in the clinic. xAARA will be explicitly positioned as an assistant, not an authority, and will surface automated assessments that help clinicians adapt therapy in real time. It will also support the integration of assessments and adaptations into the patient record for further analysis. As xAARA matures, we hope to adapt it for the automated assessment of therapy sessions in the home, where the system will provide summaries of patient progress to the therapist and allow the therapist to remotely review progress, adapt the schedule of exercises for the patient, and offer remote input on movement elements that the patient can focus on during therapy. We also plan to test the current ARAT assessment tool in other clinics as an assessment assistant and/or as a therapist training tool. Across these applications, we plan to measure convergence speed and final DBN quality relative to networks initialized from scratch.

In parallel, we will begin testing the porting of the methodology to educational and surgical contexts. Through these diverse applications, we hope to publish a portable “*porting protocol*” (vocabulary elicitation, view/interaction mapping, capture constraints, uncertainty audits, and network transfer guidelines) that enables new sites and tasks to leverage existing validated networks while adapting them to local contexts. The long-term vision is a library of pretrained DBN networks for families of related complex tasks, enabling rapid deployment of human-AI collaboration systems that inherit accumulated knowledge while remaining open to domain-specific adaptation.

## Conclusion

We proposed a theory and a related methodology for designing and implementing human-AI collaboration in the performance of complex tasks. The theory differentiates human embodied intelligence from computational intelligence and hypothesizes opportunities for synergy in which artificial intelligence enhances human performance rather than attempting to replicate or replace it. The methodology leverages observable elements of human expert performance on complex tasks to build a computational ensemble for human-AI collaboration. At the core of this ensemble is a bidirectional Dynamic Bayesian Network (DBN) that computes a nested network representation (Environment, Activity, Goals, Meaning) across multiple temporal scales—from individual instants to actions, complete performances, and sequences of performances. The ensemble enhances explicit observation and analysis of complex task performance and provides recommendations that fit readily into the expert performer's workflow, reduce entropy, and increase performance confidence. The expert performer decides whether and how to apply the recommendations in their practice. Expert validation is fed back into the computational ensemble, improving computational insights and permanently updating the network structure. The methodology was successfully implemented for human-AI collaboration in physical therapy assessment. The methodology is portable to other experts' settings. Planned applications include transdisciplinary educational research (modeling student learning experiences) and surgical outcome prediction (real-time decision support). By combining an interpretable probabilistic translator (DBN) with targeted predictive modules (AI agents) and human embodied intelligence, this theory and methodology offer a practical path to developing AI assistants that are expert-aligned, auditable, and that become more confident—and more useful—with experience. AI is thus recontextualized as a tool to enhance human potential.

## Data Availability

The raw data supporting the conclusions of this article will be made available by the authors, without undue reservation.

## References

[B1] Ackerman D. (1991). A Natural History of the Senses. New York, NY: Vintage.

[B2] Ahlheim C. Stadler W. Schubotz R. I. (2014). Dissociating dynamic probability and predictability in observed actions—an fMRI study. Front. Hum. Neurosci. 8:273. doi: 10.3389/fnhum.2014.00273 24847235 PMC4019881

[B3] Ahmed T. Rikakis T. Kelliher A. Wolf S. L. (2024). A hierarchical Bayesian model for cyber-human assessment of movement in upper extremity stroke rehabilitation. IEEE Trans. Neural Syst. Rehabil. Eng. 32, 3157–3166. doi: 10.1109/TNSRE.2024.3450008 39186425

[B4] Ahmed T. Thopalli K. Rikakis T. Turaga P. Kelliher A. Huang J.-B. . (2021). Automated movement assessment in stroke rehabilitation. Front. Neurol. 12:720650. doi: 10.3389/fneur.2021.720650 34489855 PMC8417323

[B5] ALPA (2025). Air Line Pilots Association, International. ALPA. Available online at: https://www.alpa.org (Accessed February 3, 2026).

[B6] Barsalou L. W. Niedenthal P. M. Barbey A. K. Ruppert J. A. (2003). Social embodiment. Psychol. Learn. Motiv. 43, 43–92. doi: 10.1016/S0079-7421(03)01011-9

[B7] Benner P. (2004). Using the dreyfus model of skill acquisition to describe and interpret skill acquisition and clinical judgment in nursing practice and education. Bull. Sci. Technol. Soc. 24, 188–199. doi: 10.1177/0270467604265061

[B8] Bernstein L. (1975). Leonard Bernstein Discussing Beethoven's 6th and 7th Symphony. Available online at: https://www.youtube.com/watch?v=OuYY1gV8jhU (YouTube video) (Accessed February 3, 2026).

[B9] Brown B. Reeves S. Sherwood S. (2011). “Into the wild: challenges and opportunities for field trial methods,” in Proceedings of the SIGCHI Conference on Human Factors in Computing Systems (New York, NY: ACM), 1657–1666. doi: 10.1145/1978942.1979185

[B10] Burkholder J. P. Grout D. J. Palisca C. V. (2006). A History of Western Music. New York, NY: WW Norton.

[B11] Buschman T. J. Miller E. K. (2014). Goal-direction and top-down control. Philos. Trans. R. Soc. B: Biol. Sci. 369:20130471. doi: 10.1098/rstb.2013.0471 25267814 PMC4186225

[B12] Casey T. (2013). Reflective practice in legal education: the stages of reflection. Clin. L. Rev. 20:317.

[B13] Clayton M. Leante L. (2013). “Embodiment in music performance,” in Experience and Meaning in Music Performance (Oxford: Oxford University Press). doi: 10.1093/acprof:oso/9780199811328.003.0009

[B14] Cooper A. B. Tisdell E. J. (2020). Embodied aspects of learning to be a surgeon. Med. Teach. 42, 515–522. doi: 10.1080/0142159X.2019.1708289 31944141

[B15] Darbellay F. (2015). Rethinking inter-and transdisciplinarity: undisciplined knowledge and the emergence of a new thought style. Futures 65, 163–174. doi: 10.1016/j.futures.2014.10.009

[B16] Dave D. M. Mandvikar S. Engineer P. A. (2023). Augmented intelligence: human-AI collaboration in the era of digital transformation. Int. J. Eng. Appl. Sci. Technol. 8, 24–33. doi: 10.33564/IJEAST.2023.v08i06.003

[B17] Dewey J. (2024). “Art as experience,” in Anthropology of the Arts (London: Routledge), 37–45. doi: 10.4324/9781003578123-6

[B18] Dourish P. (2001). Where the Action is: the Foundations of Embodied Interaction. Cambridge, MA: MIT press. doi: 10.7551/mitpress/7221.001.0001

[B19] Ehrenfeld J. M. (2022). Artificial intelligence versus augmented intelligence: what's in a word? Biomed. Instrum. Technol. 56, 130–131. doi: 10.2345/0899-8205-56.4.130

[B20] FAA (2025). Fligth Manuals and Handbooks. Available online at: https://www.faa.gov/regulations_policies/handbooks_manuals/aviation (Accessed February 3, 2026).

[B21] Fooken J. Baltaretu B. R. Barany D. A. Diaz G. Semrau J. A. Singh T. . (2023). Perceptual-cognitive integration for goal-directed action in naturalistic environments. J. Neurosci. 43, 7511–7522. doi: 10.1523/JNEUROSCI.1373-23.2023 37940592 PMC10634571

[B22] Granato G. Baldassarre G. (2024). Bridging flexible goal-directed cognition and consciousness: the goal-aligning representation internal manipulation theory. Neural Netw. 176:106292. doi: 10.1016/j.neunet.2024.106292 38657422

[B23] Hollan J. Hutchins E. Kirsh D. (2000). Distributed cognition: toward a new foundation for human-computer interaction research. ACM Trans. Comput.-Hum. Interact. 7, 174–196. doi: 10.1145/353485.353487

[B24] Hutchins E. (1995). Cognition in the Wild. Cambridge, MA: MIT press. doi: 10.7551/mitpress/1881.001.0001

[B25] Iida F. Giardina F. (2023). On the timescales of embodied intelligence for autonomous adaptive systems. Ann. Rev. Control Robot. Auton. Syst. 6, 95–122. doi: 10.1146/annurev-control-063022-094301

[B26] Johnson M. (2024). The Body in the Mind: The Bodily Basis of Meaning, Imagination, and Reason. Chicago, IL: University of Chicago press.

[B27] Kelliher A. Zilevu S. Rikakis T. Ahmed T. Truong Y. Wolf S. L. . (2020). “Towards standardized processes for physical therapists to quantify patient rehabilitation,” in Proceedings of the 2020 CHI Conference on Human Factors in Computing Systems (New York. NY: ACM), 1–13. doi: 10.1145/3313831.3376706

[B28] Kelly M. Ellaway R. Scherpbier A. King N. Dornan T. (2019). Body pedagogics: embodied learning for the health professions. Med. Educ. 53, 967–977. doi: 10.1111/medu.13916 31216603

[B29] Kim H. S. (1999). Critical reflective inquiry for knowledge development in nursing practice. J. Adv. Nurs. 29, 1205–1212. doi: 10.1046/j.1365-2648.1999.01005.x 10320505

[B30] Korteling J. E. van de Boer-Visschedijk G. C. Blankendaal R. A. Boonekamp R. C. Eikelboom A. R. (2021). Human-versus artificial intelligence. Front. Artif. Intell. 4:622364. doi: 10.3389/frai.2021.622364 33981990 PMC8108480

[B31] Koshy K. Limb C. Gundogan B. Whitehurst K. Jafree D. J. (2017). Reflective practice in health care and how to reflect effectively. IJS Oncol. 2:e20. doi: 10.1097/IJ9.0000000000000020 29177215 PMC5673148

[B32] Krakauer D. C. (2024). The Complex World. Santa Fe, NM: Santa Fe Institute Press. doi: 10.37911/9781947864627

[B33] Krakauer J. W. (2006). Motor learning: its relevance to stroke recovery and neurorehabilitation. Curr. Opin. Neurol. 19, 84–90. doi: 10.1097/01.wco.0000200544.29915.cc 16415682

[B34] Lee J. Ahmed T. Rikakis T. Turaga P. (2025). “Automatic temporal segmentation for post-stroke rehabilitation: a keypoint detection and temporal segmentation approach for small datasets,” in Proceedings of the Winter Conference on Applications of Computer Vision (Tucson, AZ: IEEE), 21–29. doi: 10.1109/WACVW65960.2025.00008

[B35] Mantzoukas S. Jasper M. A. (2004). Reflective practice and daily ward reality: a covert power game. J. Clin. Nurs. 13, 925–933. doi: 10.1111/j.1365-2702.2004.01008.x 15533098

[B36] Maturana H. R. Varela F. J. (2012). Autopoiesis and Cognition: The Realization of the Living, Vol. 42. Cham: Springer Science & Business Media.

[B37] McAdams S. E. Bigand E. E. (1993). “Thinking in sound: the cognitive psychology of human audition,” in Based on the fourth workshop in the Tutorial Workshop series organized by the Hearing Group of the French Acoustical Society. Oxford: Clarendon Press/Oxford University Press. doi: 10.1093/acprof:oso/9780198522577.001.0001

[B38] McDonnell M. (2008). Action research arm test. Aust. J. Physiother. 54:220. doi: 10.1016/S0004-9514(08)70034-5 18833688

[B39] Merleau-Ponty M. Landes D. Carman T. Lefort C. (2013). Phenomenology of Perception. London: Routledge. doi: 10.4324/9780203720714

[B40] Miller J. H. Page S. E. (2009). Complex Adaptive Systems: An Introduction to Computational Models of Social Life: An Introduction to Computational Models of Social Life. Princeton, NJ: Princeton University Press. doi: 10.1515/9781400835522

[B41] Monteith S. Glenn T. Geddes J. R. Achtyes E. D. Whybrow P. C. Bauer M. . (2024). Differences between human and artificial/augmented intelligence in medicine. Comput. Hum. Behav. Artif. Hum. 2:100084. doi: 10.1016/j.chbah.2024.100084

[B42] Moravec H. (1988). Mind Children: The Future of Robot and Human Intelligence. Cambridge, MA: Harvard University Press.

[B43] National Careers Service (2025). Airline Pilot Careers. Available online at: https://nationalcareers.service.gov.uk/job-profiles/airline-pilot (Accessed February 3, 2026).

[B44] Newell A. Rosenbloom P. S. Laird J. E. (1989). “Symbolic architectures for cognition,” in Foundations of Cognitive Science ed. M. I. Posner (Cambridge, MA: MIT Press), 93–131.

[B45] Osiurak F. Navarro J. Reynaud E. (2018). How our cognition shapes and is shaped by technology: a common framework for understanding human tool-use interactions in the past, present, and future. Front. Psychol. 9:293. doi: 10.3389/fpsyg.2018.00293 29563891 PMC5845892

[B46] Peña A. (2010). The dreyfus model of clinical problem-solving skills acquisition: a critical perspective. Med. Educ. Online 15:4846. doi: 10.3402/meo.v15i0.4846 20563279 PMC2887319

[B47] Piaget J. (1978). Piaget's Theory of Intelligence, Vol. 2. Englewood Cliffs, NJ: Prentice Hall.

[B48] Polanyi M. (2012). Personal Knowledge. London: Routledge. doi: 10.4324/9780203442159

[B49] Proverbio A. M. Valtolina M. (2025). Musical expertise modulates embodied processing of biological motion and audiovisual-motor integration in rhythmic hand tapping. NeuroImage 315:121287. doi: 10.1016/j.neuroimage.2025.121287 40414581

[B50] Prudkov P. (2025). On the construction of artificial general intelligence based on the correspondence between goals and means. Front. Artif. Intell. 8:1588726. doi: 10.3389/frai.2025.1588726 40607451 PMC12213789

[B51] Pylyshyn Z. W. (1988). Computing in Cognitive Science. London, ON: University of Western Ontario, Centre for Cognitive Science London. doi: 10.7551/mitpress/3072.003.0004

[B52] Sch'´on D. A. (2017). The Reflective practitioner: How Professionals Think in Action. London: Routledge.

[B53] Sergeeva A. V. Faraj S. Huysman M. (2020). Losing touch: an embodiment perspective on coordination in robotic surgery. Organ. Sci. 31, 1248–1271. doi: 10.1287/orsc.2019.1343

[B54] Simon H. A. (1990). “Bounded rationality,” in Utility and Probability, eds. J. Eatwell, M. Milgate, and P. Newman (Cham: Springer), 15–18. doi: 10.1007/978-1-349-20568-4_5

[B55] Simon H. A. (2019). The Sciences of the Artificial, Reissue of the Third Edition with a New Introduction by John Laird. Cambridge, MA: MIT press.

[B56] Sloboda J. A. (1986). The Musical Mind: The Cognitive Psychology of Music. Oxford: Oxford University Press. doi: 10.1093/acprof:oso/9780198521280.001.0001

[B57] Spreng R. N. Stevens W. D. Chamberlain J. P. Gilmore A. W. Schacter D. L. (2010). Default network activity, coupled with the frontoparietal control network, supports goal-directed cognition. Neuroimage 53, 303–317. doi: 10.1016/j.neuroimage.2010.06.016 20600998 PMC2914129

[B58] Taylor C. (1992). The Ethics of Authenticity. Cambridge, MA: Harvard University Press. doi: 10.4159/9780674237117

[B59] Tenenbaum J. B. Kemp C. Griffiths T. L. Goodman N. D. (2011). How to grow a mind: Statistics, structure, and abstraction. Science 331, 1279–1285. doi: 10.1126/science.1192788 21393536

[B60] Varela F. J. Thompson E. Rosch E. (2017). The Embodied Mind, Revised Edition: Cognitive Science and Human Experience. Cambridge, MA: MIT press. doi: 10.7551/mitpress/9780262529365.001.0001

[B61] Vygotsky L. S. Cole M. (1978). Mind in Society: Development of Higher Psychological Processes. Cambridge, MA: Harvard university press.

[B62] Winstein C. J. Stein J. Arena R. Bates B. Cherney L. R. Cramer S. C. . (2016). Guidelines for adult stroke rehabilitation and recovery: a guideline for healthcare professionals from the american heart association/american stroke association. Stroke 47, e98–e169. doi: 10.1161/STR.0000000000000098 27145936

[B63] Wolpert D. M. (2007). Probabilistic models in human sensorimotor control. Hum. Mov. Sci. 26, 511–524. doi: 10.1016/j.humov.2007.05.005 17628731 PMC2637437

[B64] Yozbatiran N. Der-Yeghiaian L. Cramer S. C. (2008). A standardized approach to performing the action research arm test. Neurorehabil. Neural Repair 22, 78–90. doi: 10.1177/1545968307305353 17704352

