# Supplementary material for: A methodology for integrating AI into embodied human intelligence for the performance of complex tasks
Source: Front Artif Intell. 2026 Mar 4;9:1715898. doi: 10.3389/frai.2026.1715898 (PMC12997122; doi:10.3389/frai.2026.1715898)
Supplement: Supplementary file 1 [file Data_Sheet_1.pdf]

./logo1-eps-converted-to.pdf

Supplementary Material

1 SUPPLEMENTARY DATA

Table S1: Mathematical notation used in the paper.

**Notation**	**Definition / Meaning**
E, A, G, M	Four symbolic layers: *Environment* (E), *Activity* (A), *Goals* (G), *Meaning* (M).
$L \in \{E, A, G, M\}$	Index over layers.
Σ_t^L	Vocabulary/lexicon of explicit symbols in layer L at instant t.
$s^L \in \Sigma^L$	A concrete symbol in layer L.
S_t^L	Random variable for the symbol realized in layer L at instant t.
Obs_t^L	Observations (from capture/extractors) used to infer S_t^L under Σ_t^L.
$\widetilde{\mathcal{S}}_t^L$	Proposed (candidate) new codes for layer L at instant t.
$u_t^L(s) \in \{0, 1\}$	Discovery/gating decision for proposal $s \in \widetilde{\mathcal{S}}_t^L$ (1=accept).
$\mathcal{R}_t^{c4}$	Set of active cross–layer $c4$ edges (quadruples) at instant t.
$r = (s^E, s^A, s^G, s^M)$	Candidate $c4$ *edge* (quadruple of layer symbols).
$\kappa_t(r) \in [0, 1]$	Compatibility potential for quadruple r at instant t (higher = more compatible).
$\tau_t \in (0, 1)$	Time–varying threshold used in $c4$ activation (explore $\to$ exploit schedule).
$\beta > 0$	Sharpness parameter of the $c4$ activation sigmoid.
$\sigma(x) = \frac{1}{1+e^{-x}}$	Logistic (sigmoid) function used in the $c4$ builder and the acceptance gate.
$\pi_t^{\mathrm{coh}}(r)$	Coherence *mixture prior* over edges r at instant t (action/task/sequence mixture).
$\psi_t^{\mathrm{coh}}(r)$	Coherence *log–odds* for edge r at instant t; injected into the $c4$ builder.
$C_{k,\le t-1}^{\mathrm{act}}(r)$	Prefix count of edge r within action k up to $t-1$ (similarly for task, sequence).
$N_{k,\le t-1}^{\mathrm{act}}$	Total count at the action level (similarly $N_{T,\le t-1}^{\mathrm{task}}$, $N_{\rho,\le t-1}^{\mathrm{seq}}$).
$b(r)$	Normalized back–off prior over edges r used for Dirichlet (add–α) smoothing.
$\alpha > 0$	Add–α (Dirichlet) smoothing strength for histogram probabilities.
$w_{\mathrm{act}}, w_{\mathrm{task}}, w_{\mathrm{seq}}$	Mixture weights for coherence prior across action, task, sequence (sum to 1).
$k(t)$, $T(t)$, $\rho(t)$	Indices of the current action, task (performance), and sequence at instant t.
$\mathcal{I}_k$, $\mathcal{U}_T$, T_ρ	Index sets: instants in action k; actions in task T; tasks in sequence ρ.
H_t	Instantaneous observable state tuple $(S_t^E, S_t^A, S_t^G, S_t^M, \mathcal{R}_t^{c4})$.
H_k^{act}, H_T^{task}, H_ρ^{seq}	Aggregated summaries at action, task, and sequence levels.
$\mathrm{Agg}(\cdot)$, $\mathrm{Compose}(\cdot)$	Operators to pool instants into action summaries and compose them upward.

Notation	Definition / Meaning
$\Phi_{\leq t-1}$	Fixed–size features distilled from $\{H_{\cdot}^{\text{act}}, H_{\cdot}^{\text{task}}, H_{\cdot}^{\text{seq}}\}$ up to $t-1$.
H_{t-1}	History up to $t-1$ (including posterior over $(g,m)_{t-1}$ and $\Phi_{\leq t-1}$).
(g_t, m_t)	Latent *orientation*: goal (g_t) and meaning/context (m_t) at instant t.
$p_{\downarrow}$	Top–down transition prior over orientation, computed before observing time-t evidence.
$a_t \in \mathcal{A}$	Action chosen at instant t by the search/attention policy.
π_{SEARCH}	Search/attention policy (e.g., EIG minus cost).
$\text{IG}_t(a)$, $\text{cost}(a)$	Action utility terms: information gain about (g,m) and action cost.
$p(S_t^L \mid \text{Obs}_t^L, \Sigma_t^L)$	Per–layer symbolization likelihoods for $L \in \{E, A, G, M\}$.
$p(\mathcal{R}_t^{c4} \mid .)$	Likelihood for which $c4$ edges are active at t (the $c4$ builder).
$p_{\uparrow}$	Bottom–up posterior kernel to update orientation with current evidence.
L_t	Step–t likelihood block: product of per–layer symbolization likelihoods and $c4$ builder term.
$p_t(\cdot)$	Step–t joint posterior over orientation, symbols, and $c4$ edges, given realized observations.
$\tilde{p}_t((g,m)_t)$	Predictive prior for $(g,m)_t$ by averaging $p_{\downarrow}$ over the previous posterior.
$q_t(r)$	Instantaneous edge distribution over $c4$ edges.
H_t^{c4}	Entropy of the instantaneous $c4$ edge distribution $q_t(r)$.
C_t	Instantaneous coherence score: expected $\psi_t^{\text{coh}}(r)$ under q_t.
$\Delta C_L^{\text{agg}}(s)$	Aggregate coherence improvement after adding symbol s to layer L (weighted across act/task/sequence).
$\text{nGain}_{c4}^L(s)$	Normalized visibility gain in expected $\lvert\mathcal{R}_t^{c4}\rvert$ from adding symbol s to layer L.
$\lambda_{\text{coh}}, \lambda_{\text{vis}}, \lambda_{\text{val}}$	Weights in the discovery gate for coherence, visibility, and expert–validation terms.
val_{c4}^L	Expert validation term used in the discovery gate for recommendations in layer L.
Λ_t	Lexicon/configuration state at t (current $\Sigma_t^{\bullet}$ and related calibration/compatibility params).
$\bar{P}(\tilde{Y}_j)$, $\bar{p}(m_{j,q}{=}1)$	Fused ensemble posteriors (logit–space fusion) for ordinal sub–action score and MQE presence.
G_j	Sub–goal code attached to sub–action j.
$\hat{m}_{t,j,q}$	Per–instant soft evidence for MQE q at sub–action j (projected from ensemble).
$\varphi_0(\cdot)$, $\varphi_{\text{mqe}}(\cdot)$, $\varphi_{\text{view}}(\cdot)$	Log–linear components of the compatibility potential (main, MQE, view).
Σ_t^{kin}	Kinematics features/summary at instant t used in compatibility.
λ_{focus}, $\pi_{\text{focus}}(q \mid s_t^A)$	Focus prior and its weight for MQE q given activity symbol s_t^A (ARAT instantiation).
w_{tr}, $w_{\text{hbm}} \geq 0$	Fusion weights for transformer and HBM modules in logit–space fusion.
$\pi_t \in [0,1]$, $s(\cdot)$, $p \geq 1$	Progress within the current action, scheduling function, and exponent used in τ_t scheduling.
